# Constrained Nonlinear and Mixed Effects Integral Differential Equation Models for Dynamic Cell Polarity Signaling

**DOI:** 10.3389/fpls.2022.847671

**Published:** 2022-05-25

**Authors:** Zhen Xiao, Nicolas Brunel, Chenwei Tian, Jingzhe Guo, Zhenbiao Yang, Xinping Cui

**Affiliations:** ^1^Department of Statistics, University of California, Riverside, Riverside, CA, United States; ^2^Laboratoire de Mathématiques et Modélisation d'Evry, UMR CNRS 8071, ENSIIE, Évry-Courcouronnes, France; ^3^Department of Botany and Plant Sciences, University of California, Riverside, Riverside, CA, United States; ^4^Institute for Integrative Genome Biology, University of California, Riverside, Riverside, CA, United States

**Keywords:** cell polarity, constrained semiparametric regression, identifiability, integro-differential equation, method of moments, semilinear elliptic equation

## Abstract

Polar cell growth is a process that couples the establishment of cell polarity with growth and is extremely important in the growth, development, and reproduction of eukaryotic organisms, such as pollen tube growth during plant fertilization and neuronal axon growth in animals. Pollen tube growth requires dynamic but polarized distribution and activation of a signaling protein named ROP1 to the plasma membrane *via* three processes: positive feedback and negative feedback regulation of ROP1 activation and its lateral diffusion along the plasma membrane. In this paper, we introduce a mechanistic integro-differential equation (IDE) along with constrained semiparametric regression to quantitatively describe the interplay among these three processes that lead to the polar distribution of active ROP1 at a steady state. Moreover, we introduce a population variability by a constrained nonlinear mixed model. Our analysis of ROP1 activity distributions from multiple pollen tubes revealed that the equilibrium between the positive and negative feedbacks for pollen tubes with similar shapes are remarkably stable, permitting us to infer an inherent quantitative relationship between the positive and negative feedback loops that defines the tip growth of pollen tubes and the polarity of tip growth.

## 1. Introduction

Cell polarity describes the asymmetric property of a cell, a fundamental feature of almost all cells. It is required for the differentiation of new cells, cell shape formation, polar cell growth, cell migration, etc. A well-known example of cell polarity in plants is found in the polar growth of pollen tubes (termed tip growth), which delivers sperms to the ovary for fertilization. Pollen tubes are one of the fastest growing cells in plants and therefore represent an attractive model system to investigate polarized cell growth (Yang, 1998, 2008; Hepler et al., 2001; Lee and Yang, 2008; Qin and Yang, 2011; Luo et al., 2017; Guo and Yang, 2020). Similar polar cell growth is also found in fungi and animals, such as fungal hyphal growth and neunronal axon extension (Gow, 1994; Palanivelu and Preuss, 2000; Gow et al., 2002; Wen and Zheng, 2006; Lowery and Vanvactor, 2009; Takano et al., 2019; Bassilana et al., 2020).

**Figure 1:**
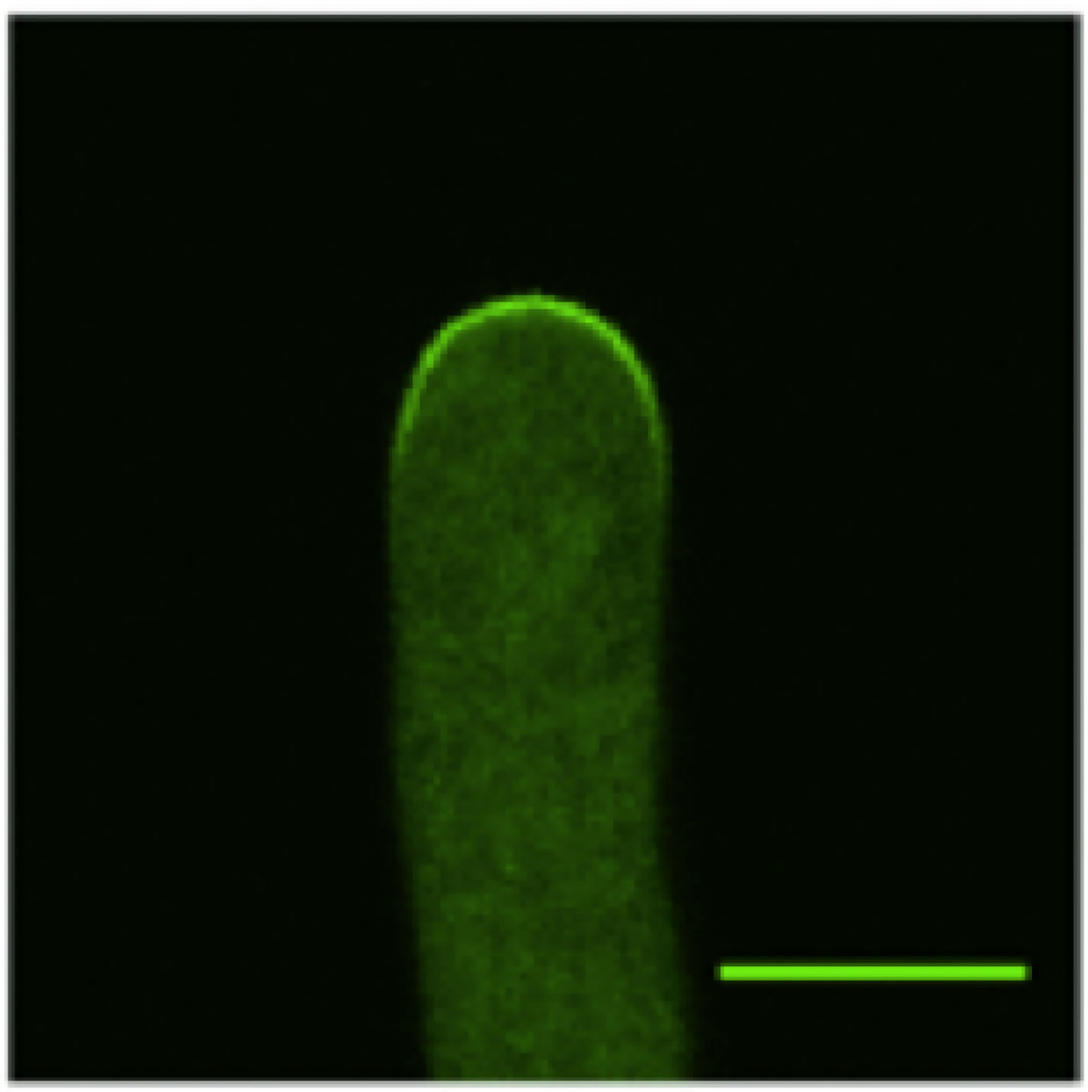
Confocal microscopy image of a wild-type *Arabidopsis* pollen tube expressing CRIB4-GFP that shows the distribution of the active ROP1. Only the tip region of the pollen tube is shown (The bar is 7 μm).

**Figure 2:**
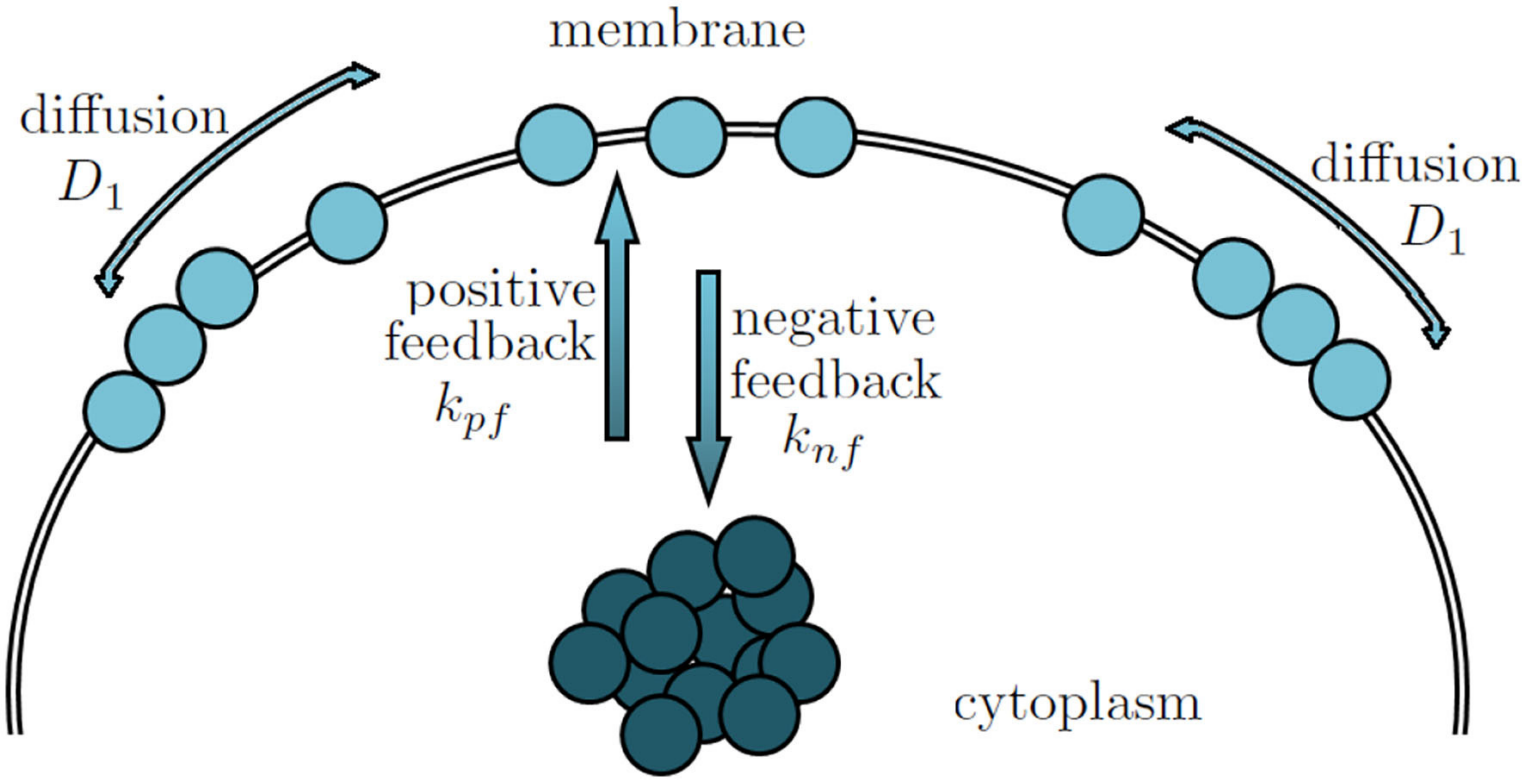
The formation of ROP1 polarity is determined by the positive, negative feedbacks and the lateral diffusion.

Several mathematical models have been developed to simulate pollen tube tip growth (Dumais et al., 2006; Kroeger et al., 2008; Campas and Mahadevan, 2009; Lowery and Vanvactor, 2009; Fayant et al., 2010). These models focused on the cell wall mechanics and the cell wall mechanics-mediated shape formation of pollen tubes. However, it has been demonstrated that ROP1, a pollen-specific member of the ROP subfamily of Rho GTPases, is a central regulator of pollen tube tip growth (Gu et al., 2003). In particular, it has been found that the local concentration of active ROP1 on the plasma membrane (as shown in Figure 1) plays a predominant role in determining the polarity of the pollen tubes (Lin et al., 1996; Li et al., 1998). Hwang et al. (2010) suggested that the distribution of active ROP1 is determined by three processes: ROP1 activation through positive feedback, deactivation through negative feedback, and diffusion (as shown in Figure 2).

Luo et al. (2017) proposed a model of pollen tube tip growth that consists of two parts: the exocytosis-ROP1 polarization (ERP) module and the Exocytosis-Wall Extension module. In the ERP module, they proposed a system of evolutionary partial differential equation (PDE) to simulate the spatiotemporal dynamics of active ROP1 determined by the aforementioned three processes and to predict how the shape of pollen tubes changes when there is a change in positive feedback strength, negative feedback strength, or degrees of deficiency in exocytosis that regulates both positive feedback and negative feedback. Altering either one can result in pollen tubes with different widths (refer to Luo et al., 2017; Figures 1, 2 and Supplementary Figure 3). For example, a mutant with a loss of ROP1 deactivator REN1 is predicted to have an 80% reduction in negative feedback strength and therefore produces a wider tip and makes slower and smoother turnings to the guidance signal when compared to a wild type (Luo et al., 2017; Figure 1b). A weak mutant for an exocyst subunit gene SEC8 treated with 50 nM of Latrunculin B is predicted to have 43% reduction in exocytosis and therefore shows a broader distribution of active ROP1 and increased tube width when compared to a wild type. The strengths of positive and negative feedback loops in ERP module were not measurable and thus were computed using a trial-and-error method so that active ROP1 distribution can be simulated based on their evolutionary PDE model. As they pointed out, however, constructing a realistic mathematical model for pollen tube tip growth with testable and robust predictive powers requires accurately determining the strengths of positive and negative feedback loops.

Following Luo et al. (2017), we propose a similar integro-differential equation (IDE) model to describe the interplay among the aforementioned three processes that lead to ROP1 polarity formation at a steady state. Different from Luo et al. (2017), we devote our effort to estimating the strengths of positive feedback and negative feedback in the IDE model based on steady state ROP1 data. Standard parameter estimation for ordinary differential equation (ODE), such as gradient matching or generalized profiling (Ramsay et al., 2007; Brunel, 2008; Wu and Chen, 2008; Brunel et al., 2014), might be adapted to IDE under appropriate regularity assumptions (Lakshmikantham, 1995), but with challenging two identifiability issues (Miao et al., 2011). One is whether the solution of the nonlinear IDE exists and is unique (Gutenkunst et al., 2007; Transtrum et al., 2011). The other is whether the observed data are sufficient to estimate the parameters in the model (related to the Fisher Information Matrix).

Integro-differential equations of positive integer order have been widely used in many scientific areas (Bohner et al., 2021), and it is very important to investigate the qualitative properties of solutions (Tunç and Tunç, 2018). Recently, Tunç and Tunç (2018) presented sufficient and necessary conditions on the stability of the solutions to the non-linear scalar Volterra IDE and Volterra integro-differential systems of the first order. They further studied the qualitative properties of solutions to nonlinear Volterra IDE with Caputo fractional derivative, multiple kernels, and multiple constant delays and derived new sufficient conditions related to the stabilities and boundedness of solutions (Bohner et al., 2021).

Recently, there are growing applications of fractional differential equations and fractional integro differential equations in various fields such as engineering (Baleanu et al., 2020a; Bohner et al., 2021; Thabet et al., 2021) and biology (Baleanu et al., 2020c; Mohammadi et al., 2021). The Caputo and Riemann-Liouville Derivatives are the two most famous fractional derivatives that are introduced in order to take into account non-locality behavior in the phenomena of interest. The equations involved in the papers (Aydogan et al., 2018; Baleanu et al., 2019a,b, 2020a,b,c; Alizadeh et al., 2020; Matar et al., 2021; Mohammadi et al., 2021; Thabet et al., 2021) are mixed relations between integral and derivatives of the functions. While all these papers successfully addressed the critical problem of proving the existence and uniqueness of a solution to these new models, it also shows that general results for existence and uniqueness in IDE are still not found, and a careful and adapted proof should be derived on a case by case basis. The papers mentioned above use a variety of different techniques for constructing the solution: for instance, Laplace Transforms and Picard iterations.

In this paper, we exploit the closeness of our IDE to the semilinear equation, in order to apply the results of the theory of standard semilinear PDE to our case. We provide sufficient and necessary conditions for the existence and uniqueness of the solution to the proposed IDE. Furthermore, we integrate these conditions as constraints into our new Method of Moments (Lu and Meeker, 1993) estimation procedure to allow estimating positive feedback rate and negative feedback rate in a single tube IDE model or a multiple tube IDE model. For the latter case, we naturally use the framework of mixed-models (Li et al., 2002; Putter et al., 2002; Huang and Wu, 2006; Guedj et al., 2007; Lu et al., 2011) for estimating the population parameters, but we avoid the use of the likelihood. The corresponding procedure is equivalent to estimating a mixed-effects ODE model with linear constraints. We show that the estimators are consistent with asymptotic distributions under general conditions. We also prove that the two parameters are identifiable given the data.

While we could possibly incorporate fractional derivatives in our model, such modifications of our model are not necessary at this stage because we already have a very good agreement between model predictions and data. Moreover, we dedicate a large portion of the paper to measuring the population variation and the observation error, and our objective is to identify the stability of the balance between positive and negative feedbacks.

The paper is organized as follows. In Section 2, we describe how our IDE model is derived in order to characterize the distribution of active ROP1 on the membrane at a steady-state. We provide sufficient and necessary conditions for the existence and uniqueness of the solution to the proposed IDE model with a tractable and generic expression. We then introduce the IDE-based statistical models for a single pollen tube and for multiple pollen tubes. We estimate the individual and population parameters in both models, based on a constrained method of moments (CMM) procedure, and derive the asymptotic properties of CMM. We also prove that the two parameters are identifiable given the data. We examine the performance of the proposed estimation procedures through simulation studies and real data analysis in Section 3. The paper is concluded in Section 4.

## 2. Methods

### 2.1. An IDE Model for a Steady-State Distribution of Active ROP1

**Figure 3:**
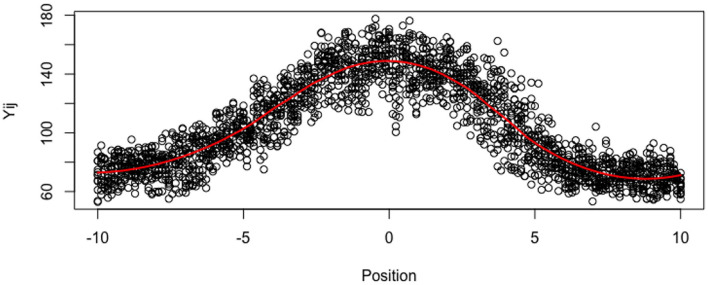
Imputated Data ỹ_*ij*_ for all tubes at all locations. The red line is loess smoother.

In this paper, we aim at deriving a mechanistic and interpretable model for distribution functions *x* ↦ *R*(*x*), defined on an interval [−*L*_0_, *L*_0_], where *L*_0_ ≤ +∞. The function *x* ↦ *R*(*x*) denotes the active ROP1 concentration at the location *x* on the membrane, and the function is centered such that the apical tip is obtained at the location *x* = 0 (as shown in Figure 3). The tube membrane is indexed by its curvilinear abscissa *x*, and its total length is 2 × *L*_0_. The function *R* is positive, bounded and the integral $\int_{a}^{b}R\left(x\right)dx$ is equal to the quantity of ROP1 along the segment [*a, b*], −*L*_0_ < *a* < *b* < *L*_0_. This implies that the function *R* could be almost considered a non-normalized density function.

Following the approach developed in Luo et al. (2017) and Altschuler et al. (2008), we consider a stationary PDE model that describes the equilibrium among the three competing forces that lead to ROP1 polarity formation: ROP1 activation through positive feedback with rate *k*_*pf*_ > 0, deactivation through negative feedback with rate *k*_*nf*_ > 0, and diffusion with coefficient *D*_1_ > 0 (as shown in Figure 2). A general semilinear elliptic equation with Dirichlet conditions (Cazenave and Haraux, 1998; Badiale and Serra, 2010) can be used to describe such a stationary PDE


(1) $$\begin{array}{l} \{\begin{array}{cc} & -D_{1}\partial_{x}^{2}R=-k_{nf}g_{n}\left(R\right)+k_{pf}g_{p}\left(R\right)\\ where & \;x\in \left[-L_{0},L_{0}\right],\;R\left(-L_{0}\right)=R\left(L_{0}\right)=0. \end{array} \end{array}$$

The rate of ROP1 lateral diffusion *D*_1_ on the plasma membrane can be measured by fluorescence recovery after photobleaching (FRAP) (Luo et al., 2017). *k*_*pf*_ and *k*_*nf*_ are determined by ROP1-independent constants linking ROP1 activity to the local rate of exocytosis and exocytosis-independent constants determined by the enzyme activity and expression levels of GEF and GAP (Luo et al., 2017). While *k*_*nf*_ and *k*_*pf*_ are not experimentally measurable, they can be estimated from observed active ROP1 concentration on the membrane at the steady state. Model (1) gives a direct measure of the relative importance of the different mechanisms in the system.

The functions *g*_*n*_ and *g*_*p*_ are positive functions that describe the laws of the underlying mechanisms. When the exact physical or chemical mechanisms are known (such as the law of mass action, or Michaelis-Menten kinetics), we can give exact functional expression. But in general, the mechanisms are often partially known and we can only consider qualitative behaviors. Typically, standard assumptions are that *g*_*n*_ and *g*_*p*_ are both smooth increasing functions. The linearity assumption *g*_*n*_(*u*) = *u* for the negative feedback is common in numerous models used in applications (Altschuler et al., 2008; Badiale and Serra, 2010; Luo et al., 2017). Remarkably, the existence and uniqueness of a positive solution (different from the trivial solution *R* = 0) is guaranteed under general conditions (Lions, 1982). In addition, under very general conditions on *g*_*p*_, this positive solution is symmetric around 0 and decreasing on [0, *L*_0_] (see Theorem 1 in Gidas et al., 1979). These results indicate that all the solutions *R* of the PDE models (1) share surprisingly the same qualitative properties: they are bell-shaped and even functions that vanish at the boundaries and therefore can characterize well the ROP1 distribution on the membrane as revealed in Figure 1. This typically reduces the interest in exploring the use of refined functions *g*_*p*_ as they will provide the same pattern for *R*. As a consequence, we concentrate on the case of a superlinear *g*_*p*_, defined as $g_{p}\left(u\right)=u^{\alpha },\alpha >1$, i.e., it grows faster than a linear function when *u* tends to infinity.

An obvious limitation of the model (1) is that the two competing positive and negative forces do not exhibit any saturation or depletion effect, as both functions *g*_*p*_ and *g*_*n*_ are assumed to be nondecreasing for any *u* > 0. For the negative feedback, it is standard to assume that the negative feedback is linear and does not depend on the actual quantity *u*. However, the monotonicity of the positive feedback is questionable. Indeed, the positive feedback is prone to be limited by the depletion of the available material. A standard assumption as used in the logistic equation (Murray, 2007) should be that the rate decreases with the ratio of available ROP1 in the cytosole, i.e., $\left(1-\frac{\int_{-L_{0}}^{L_{0}}R\left(x\right)dx}{R_{tot}}\right)$, where *R*_*tot*_ is total free ROP1 in the cell.

Putting all together, we refine the stationary PDE (1) as the following stationary IDE


(2) $$\begin{array}{l} \{\begin{array}{cc} & -D_{1}\partial_{x}^{2}R=-k_{nf}R+k_{pf}\left(1-\frac{\int_{-L_{0}}^{L_{0}}Rdx}{R_{tot}}\right)R^{\alpha }\\ where\;x\in \left[-L_{0},L_{0}\right],\;R\left(-L_{0}\right)=R\left(L_{0}\right)=0. \end{array} \end{array}$$

Here *D*_1_ is diffusion coefficient. *k*_*pf*_ and *k*_*nf*_ are positive feedback rate and negative feedback rate, respectively. α determines how faster the positive feedback function grows than the negative feedback function when the active ROP1 concentration goes to infinity.

**Remark 1:** A similar model for cell polarity was introduced in Altschuler et al. (2008), except that their model includes a spontaneous association term and α is assumed to be 1. The nonlinearity in our model comes from the product of *R*^α^ with α > 1 and the fraction of “available” ROP1 particles $1-\frac{\int_{-L_{0}}^{L_{0}}R\left(x\right)dx}{R_{tot}}$.

**Remark 2:** While the fraction $\frac{\int_{-L_{0}}^{L_{0}}R\left(x\right)dx}{R_{tot}}$ plays a prominent role in a specific dynamics driven by a particular ODE in Altschuler et al. (2008), we consider directly an IDE that describes the intertwined dynamics of *R* and $\frac{\int_{-L_{0}}^{L_{0}}R\left(x\right)dx}{R_{tot}}$.

**Remark:3** The total number of ROP1 on the membrane $\int_{-L_{0}}^{L_{0}}R\left(x\right)dx$ must be lower than the total free ROP1 *R*_*tot*_ in the cell, which will be an important constraint to satisfy in our model for the existence of a solution.

### 2.2. Identifiability of Solution *R*

For the reference nondimensionalized model (3) below, we can prove the existence and uniqueness of the positive solutions *U*_α_ and its limited sensitivity to α and *c* (see Lemma 1 in Web Appendix),


(3) $$\begin{array}{l} \{\begin{array}{cc} -\partial_{x}^{2}u=-u+u^{\alpha }, & x\in \left[-c,c\right]\\ u\left(-c\right)=u\left(c\right)=0. \end{array} \end{array}$$

As a result, there exists unique positive solution(s) *R*_λ, μ_(*x*) = λ*U*_α_(μ*x*) to the IDE (2) with $\mu =\sqrt{\frac{k_{nf}}{D_{1}}}$ and λ being the positive root(s) of the following equation


(4) $$\begin{array}{l} \frac{k_{nf}}{k_{pf}}-\lambda^{\alpha -1}+\lambda^{\alpha }\sqrt{\frac{D_{1}}{k_{nf}}}\frac{\left|U_{\alpha }\right|}{R_{tot}}=0, \end{array}$$

if and only if


(5) $$\begin{array}{l} \Lambda \left(R_{tot},D_{1},\alpha ,L_{0},k_{nf},k_{pf}\right)=\frac{k_{nf}}{k_{pf}}-\frac{1}{\alpha }{\left(\frac{\alpha -1}{\alpha }\sqrt{\frac{k_{nf}}{D_{1}}}\frac{R_{tot}}{\left|U_{\alpha }\right|}\right)}^{\alpha -1}\leq 0. \end{array}$$

See Proposition 1 in Web Appendix for details. Upon checking condition (5), the solution *R*_λ,μ_(*x*) can be obtained as follows:

1. Solve the semilinear elliptic equation (3) on $\Omega^{\prime }=\left[-L_{0}\sqrt{\frac{k_{nf}}{D_{1}}},L_{0}\sqrt{\frac{k_{nf}}{D_{1}}}\right]$ and obtain the solution *U*_α_, compute |*U*_α_|.
2. Compute the discriminant function Λ(·).
3. If Λ(·) = 0, compute $\mu =\sqrt{\frac{k_{nf}}{D_{1}}}$ and the unique root λ^*^ of Equation (4) where $\lambda^{*}=\frac{\alpha -1}{\alpha }\frac{\mu R_{tot}}{\left|U_{\alpha }\right|}$, and compute the solution $R_{\lambda^{*},\mu }\left(x\right)=\lambda^{*}U_{\alpha }\left(\mu x\right)$.
4. If Λ(·) < 0, compute $\mu =\sqrt{\frac{k_{nf}}{D_{1}}}$ and the positive roots $\lambda_{1}^{*}$ and $\lambda_{2}^{*}$ of Equation (4), and compute the solutions $R_{\lambda_{1}^{*},\mu }\left(x\right)=\lambda_{1}^{*}U_{\alpha }\left(\mu x\right)$ and $R_{\lambda_{2}^{*},\mu }\left(x\right)=\lambda_{2}^{*}U_{\alpha }\left(\mu x\right)$. Note: In practice, the solution *R* closer to the experimental data will be chosen.

By solving Equation (4), we see that if *R*_λ,μ_ is a solution to the IDE (2) and Λ < 0, we have necessarily $k_{nf}=D_{1}\mu^{2}$ and $k_{pf}=D_{1}\mu^{2}{\left(\lambda^{\alpha -1}-\lambda^{\alpha }\frac{\left|U_{\alpha }\right|}{\mu R_{tot}}\right)}^{-1}$. Consequently, with a given solution *R*_λ,μ_, we cannot recover *k*_*nf*_, *k*_*pf*_, and *R*_*tot*_, because we cannot compute *k*_*pf*_ and *R*_*tot*_ from a unique λ, μ. The biological implication is that we cannot recover the total free number of ROP1 in the cell by observing only ROP1 on the cell membrane. For this reason, we need to fix *R*_*tot*_ to be a constant. In the case of Λ = 0, we still have $k_{nf}=D_{1}\mu^{2}$ while $k_{pf}=\alpha D_{1}\mu^{2}\lambda^{1-\alpha }$ is not impacted by *R*_*tot*_ anymore.

Let $r=\int_{-L_{0}}^{L_{0}}R_{\lambda ,\mu }\left(x\right)dx/R_{tot}$ denote the fraction of active ROP1 on the membrane. The existence of positive solution *R*_λ,μ_ to IDE (2) suggests that *r*^α−1^(1 − *r*) ≤ (α − 1)^α−1^/α^α^, i.e., the fractions of active ROP1 on the membrane and inactive ROP1 in the cytosol at the steady state are controlled by the nonlinear growth of positive feedback force (determined by α) competing with the linear growth of the negative feedback force.

Without an integral part in IDE (2), the solutions are still of the form *R*_λ,μ_. The parameters *k*_*nf*_, *k*_*pf*_, and *D*_1_ in the equation $-D_{1}\partial_{x}^{2}R=-k_{nf}R+k_{pf}R^{\alpha }$ can freely vary and the shape (height) of the solution changes with $\lambda ={\left(\frac{k_{pf}}{k_{nf}}\right)}^{\frac{1}{1-\alpha }}$. The introduction of the saturation effect through the integral part in IDE (2) only changes the height of the peak λ*U*_α_(0) (see Remark 2 in Web Appendix) with $\lambda ={\left(\frac{k_{pf}}{k_{nf}}\left(1-r\right)\right)}^{\frac{1}{1-\alpha }}$. Obviously, $\frac{k_{nf}}{k_{pf}}$ is an important quantity that controls the height of the peak. Recall that $\int_{-L_{0}}^{L_{0}}R_{\lambda ,\mu }\left(x\right)dx=\frac{\lambda |U_{\alpha }|}{\mu }\leq R_{tot}$, the integral term implies that *k*_*nf*_, *k*_*pf*_, and *D*_1_ are linked together, and that link is controlled by *R*_*tot*_.

### 2.3. Parameter Estimation

Note that the diffusion coefficient *D*_1_ is a positive quantity that can be experimentally measured: *D*_1_ = 0.2μ*m* (Luo et al., 2017). In order to reduce the identifiability problem between α and μ (i.e., *k*_*nf*_, see Web Supplementary Figure 2), α is fixed at 1.2 in this paper. There is also a limited sensitivity of the solution *R* to α and *L*_0_ as discussed in Section 3. Furthermore, we cannot recover biologically the total free number of ROP1 (*R*_*tot*_) in the cell by observing only ROP1 on the cell membrane. Adding all together, *D*_1_, α, *L*_0_, and *R*_*tot*_ are all assumed to be known constants and will be dropped from Λ(·) throughout this paper, and our interest lies in the estimation of the parameters *k*_*pf*_ and *k*_*nf*_ under the constraint that the solution *R* of IDE (2) exists, i.e., Λ(*k*_*pf*_, *k*_*nf*_) ≤ 0.

In this section, we first consider estimating *k*_*nf*_ and *k*_*pf*_ in a constrained nonlinear fixed effect model using a single pollen tube data. We then further extend to estimate *k*_*nf*_ and *k*_*pf*_ in a constrained nonlinear mixed effect model using multiple pollen tube data. Proposition 2 (see Web Appendix) ensures that *k*_*nf*_ and *k*_*pf*_ can be estimated using noisy data (Miao et al., 2011) obtained either from a single pollen tube or from multiple pollen tubes.

#### 2.3.1. Single Pollen Tube and Constrained Nonlinear Fixed Effect Model

For a single pollen tube, let *Y*_*j*_ be the observed ROP1 intensity at a location *X*_*j*_ (*X*_*j*_ is randomly sampled from a distribution such as a uniform distribution) on the membrane at a steady state, and


(6) $$\begin{array}{l} Y_{j}=R\left(X_{j};k_{nf},k_{pf}\right)+\epsilon_{j}\;j=1,2,\cdots \;,n. \end{array}$$

where *R*(*X*; ·) is the solution of the IDE (2) and ϵ_*j*_ are *i*.*i*.*d*. random measurement errors following a certain distribution with mean 0 and variance σ^2^. As shown in Section 2.2, *R*(*X*; ·) exists if and only if the discriminant function Λ(*k*_*nf*_, *k*_*pf*_) is non-positive. Therefore, the IDE based model (6) is subject to the following constraints


$$\begin{array}{l} \{\begin{array}{c} \Lambda \left(k_{nf},k_{pf}\right)\leq 0,\\ k_{nf}>0,k_{pf}>0. \end{array} \end{array}$$

The constrained nonlinear model (6) can be reparametrized into the following model (7) with μ and λ subject to the linear constraints (8)


(7) $$\begin{array}{l} Y_{j}=\lambda U_{\alpha }\left(\mu X_{j}\right)+\epsilon_{j},\;j=1,2,\cdots \;,n, \end{array}$$

(8) $$\begin{array}{l} \{\begin{array}{c} \Lambda^{*}\left(\mu ,\lambda \right)=\mu R_{tot}-\lambda |U_{\alpha }|>0\\ \lambda >0\\ \mu >0 \end{array} \end{array}$$

where $\mu =\sqrt{\frac{k_{nf}}{D_{1}}}$ and λ is the root of Equation (4). See Proposition 3 in the Web Appendix for proof. The choice of λ has been discussed in Section 2.2. We can simply estimate λ and μ first and then transpose them back to *k*_*nf*_ and *k*_*pf*_.

With the observations ${\left\{y_{j}\right\}}_{j=1}^{n}$ at locations ${\left\{x_{j}\right\}}_{j=1}^{n}$ obtained from the pollen tube tip growth experiment, we propose the following constrained nonlinear least square (CNLS) estimation method.

1. Compute *U*_α_(*x*)
2. Estimate μ and λ by minimizing (9) under the constraints (8)
  (9) $$\begin{array}{l} \left(\hat{\lambda },\hat{\mu }\right)=\arg\underset{\lambda ,\mu }{\min}\overset{n}{\underset{j=1}{\sum }}{\left(y_{j}-\lambda U_{\alpha }\left(\mu x_{j}\right)\right)}^{2} \end{array}$$
3. Convert $\hat{\mu }$ and $\hat{\lambda }$ to ${\hat{k}}_{nf}$ and ${\hat{k}}_{pf}$ by
  (10) $$\begin{array}{l} \{\begin{array}{c} {\hat{k}}_{nf}=D_{1}{\hat{\mu }}^{2}\\ {\hat{k}}_{pf}=\frac{D_{1}{\hat{\mu }}^{2}}{{\hat{\lambda }}^{\alpha -1}-\frac{{\hat{\lambda }}^{\alpha }|U_{\alpha }|}{\hat{\mu }R_{tot}}} \end{array} \end{array}$$
4. Estimate σ^2^ by ${\hat{\sigma }}^{2}=\frac{1}{n-2}\sum_{j=1}^{n}{\left(y_{j}-\hat{\lambda }U_{\alpha }\left(\hat{\mu }x_{j}\right)\right)}^{2}$

In step 1, *U*_α_ can be obtained by numerically solving a boundary value problem using many methods, such as the shooting method (Soetaert, 2009; Soetaert et al., 2010), the mono-implicit Runge-Kutta (MIRK) method (Cash and Mazzia, 2005), and the collocation method (Bader and Ascher, 1987) available in the R package “bvpSolve.”

To handle the linear constraint μ*R*_*tot*_ − λ|*U*_α_| > 0 in the nonlinear least-squares minimization in the second step, we introduce a new parametrization with variable $\nu ≜\frac{\mu }{\lambda }$. As a result, the constrained minimization function is simplified as follows, which can be more efficiently solved by the Levenberg-Marquardt algorithm (Bertsekas et al., 1998), coded in the R package NLSR.


(11) $$\begin{array}{l} \{\begin{array}{c} \min_{\lambda ,\nu }\sum_{j=1}^{n}{\left(y_{j}-\lambda U_{\alpha }\left(\lambda \nu x_{j}\right)\right)}^{2}\\ \lambda >0,\nu >\frac{\left|U_{\alpha }\right|}{R_{tot}} \end{array} \end{array}$$

Among other possible choices, we propose to use the following raw estimates of the parameters as the initial values,


(12) $$\begin{array}{l} \{\begin{array}{c} \tilde{\lambda }=\frac{{ỹ}_{0}}{U_{\alpha }\left(0\right)}\\ \tilde{\mu }=\frac{\tilde{\lambda }|U_{\alpha }|}{\sum_{i=2}^{n}y_{\left(i\right)}\delta_{i}} \end{array} \end{array}$$

where ỹ_0_ is the closest observed value to *x* = 0, and δ_*i*_ = *x*_(*i*)_ − *x*_(*i*−1)_ is the step size of the sorted observations *x*_*i*_. Here, we exploit the fact *R*(0;*k*_*nf*_, *k*_*pf*_) = λ*U*_α_(0), and we estimate the integral $\int_{-L_{0}}^{L_{0}}R_{\lambda ,\mu }\left(x\right)dx=\frac{\lambda |U_{\alpha }|}{\mu }$ directly from the data by $\sum_{i=2}^{n}y_{\left(i\right)}\delta_{i}$ where *y*_(*i*)_ is the observation corresponding to *x*_(*i*)_.

Let ***θ*** = (μ, λ)^*T*^ be the parameter vector, ***θ***_0_ be the true value of ***θ***, and ${\hat{\theta }}_{n}$ be the CNLS estimator with *n* sample measurements. For the general NLS estimator, the asymptotic properties have been established by Jennrich (1969). Wang (1996) states that the constraints have no impact on the asymptotic properties, and we have a standard normal distribution. Nevertheless, for the sake of completeness, we also consider the case when the true parameter values ***θ***_0_ are on the boundary of the constrained set and give a more general result (see Proposition 4 in Web Appendix). This latter situation corresponds to the case where $R_{tot}=\int_{-L_{0}}^{L_{0}}R\left(x\right)dx$, i.e., all the available ROP1 is activated, and the strength of negative feedback *k*_*nf*_ is overwhelmed by a high *k*_*pf*_. However, this case has not been observed on real data.

#### 2.3.2. Multiple Pollen Tube and Constrained Nonlinear Mixed Effect Model

**Figure 4:**
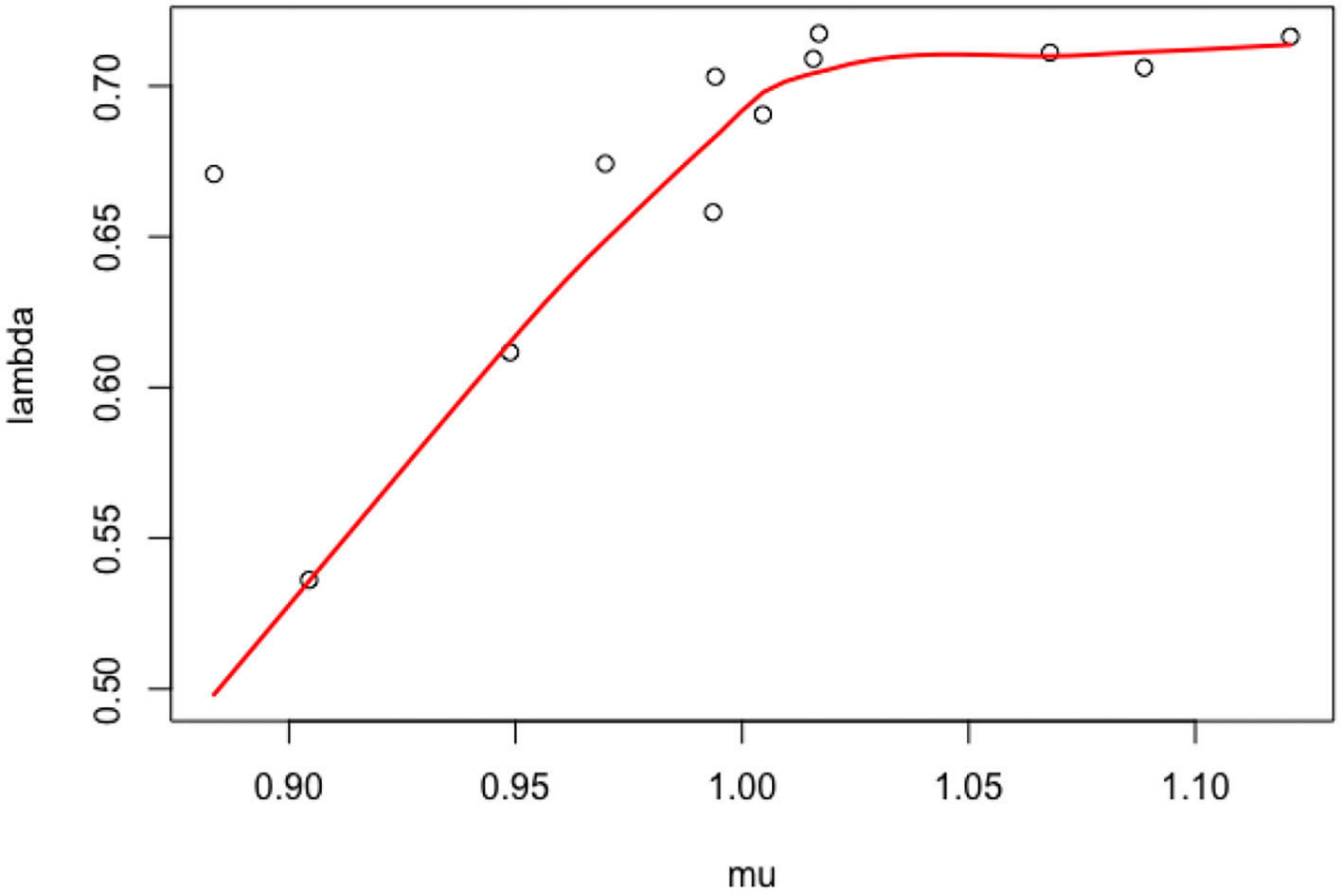
Scatter plot of the individual parameters (*k*_*nf,i*_, *k*_*pf,i*_).

**Figure 5:**
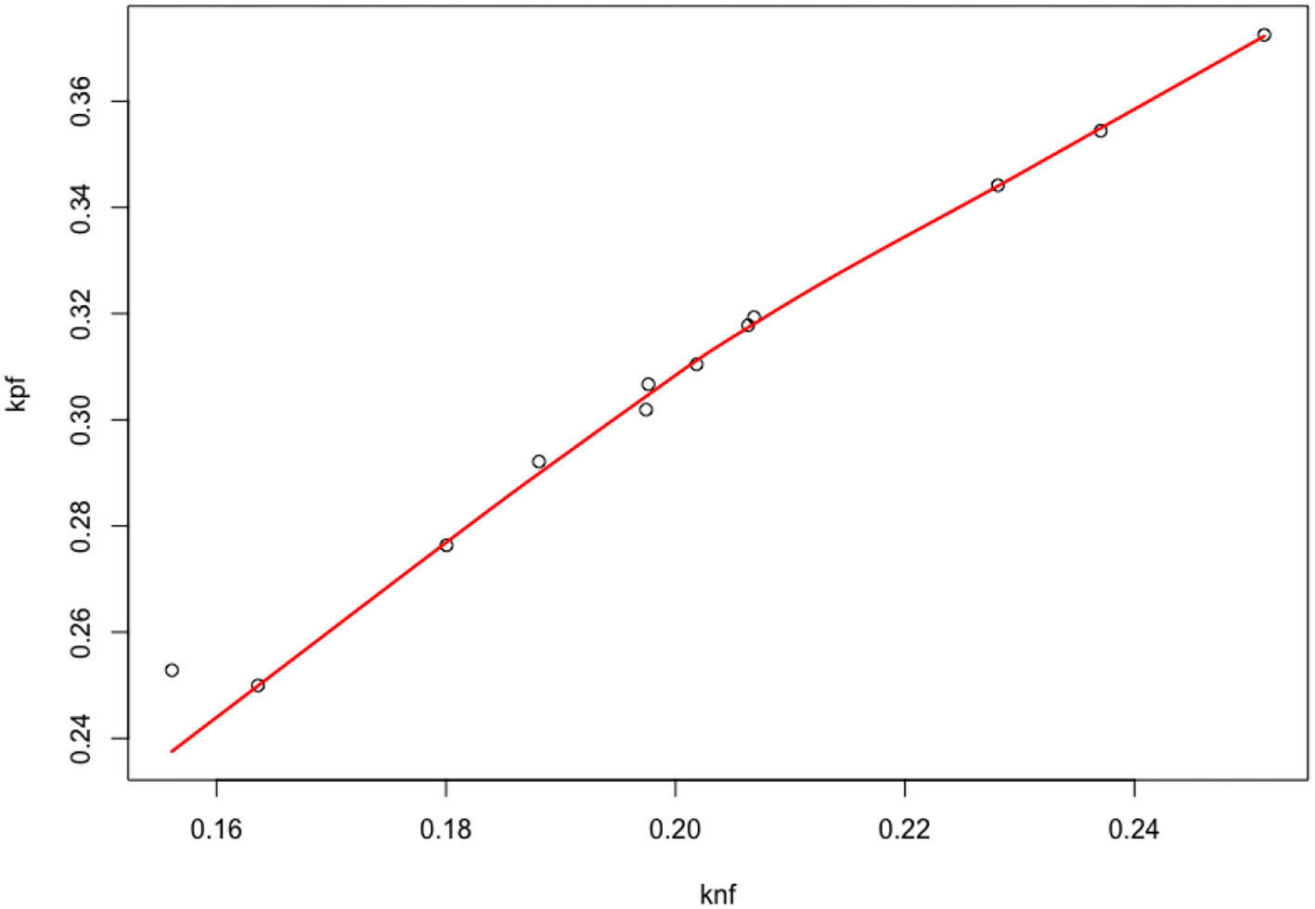
Scatter plot of the individual parameters (μ_*i*_, λ_*i*_).

As discussed in the introduction, our objective is to estimate at the population level the strength of the positive feedback and negative feedback processes that contribute to the ROP1 distribution and polarity formation at a steady state. In Section 3, we present data on ROP1 intensities collected from experiments on 12 different pollen tubes. For each pollen tube, we can fit the constrained nonlinear fixed effect model as described in Section 2.3.1 to the data associated with the tube and obtain the CNLS estimators. The estimated parameters $\left({\hat{\lambda }}_{i},{\hat{\mu }}_{i}\right),i=1,\ldots ,12$ are plotted in Figure 4. We can see some variability of λ and μ and a strong correlation between the two, and an even higher correlation between the original parameters *k*_*nf*_ and *k*_*pf*_ as shown in Figure 5. For this reason, we propose to model this individual variability with a mixed model. We extend the models (6) and (7) by considering *Y*_*ij*_, the ROP1 intensity observed for tube *i* at location *X*_*ij*_ on the membrane, as follows:


(13) $$\begin{array}{l} Y_{ij}=R_{i}\left(X_{ij};\lambda_{i},\mu_{i}\right)+\epsilon_{ij} \end{array}$$

(14) $$\begin{array}{l} =\lambda_{i}U_{\alpha }\left(\mu_{i}X_{ij}\right)+\epsilon_{ij},\;i=1,2,\cdots \;,m;j=1,2,\cdots \;,n_{i}, \end{array}$$

where ϵ_*ij*_ is *i*.*i*.*d* with mean 0 and variance σ^2^. We further assume that


(15) $$\begin{array}{l} \theta_{i}={\left(\lambda_{i},\mu_{i}\right)}^{T}\sim G\left(\theta_{0},\Sigma_{0}\right) \end{array}$$

where G is a density on the constraint set $C=\left\{\left(\lambda_{0},\mu_{0}\right)|\mu_{0},\lambda_{0}>0,\mu_{0}R_{tot}-\lambda_{0}|U_{\alpha }|>0\right\}$, such that the mean $E\left(\theta_{i}\right)=\theta_{0}={\left(\lambda_{0},\mu_{0}\right)}^{T}$ and variance *V*(***θ***_*i*_) = Σ_0_, for *i* = 1, 2, ⋯ , *m*. If there is no constraint, all the parameters can be estimated by several existing methods such as Ke and Wang (2001) and Wolfinger and Lin (1997). Due to the specific form of the constraint set C, and the absence of “natural" assumptions on the population distribution, we only assume the existence of the first two moments: the mean ***θ***_0_ and variance Σ_0_. The density G does not need to be completely specified for population inference (typically G can be a truncated Gaussian distribution on C).

Denote the experimental data to be {*y*_*ij*_} and {*x*_*ij*_} with *i* = 1, ⋯ , *m* and *j* = 1, ⋯ , *n*_*i*_. We assume that the mean ***θ***_0_ is not on the boundary (i.e., the individual parameters ***θ***_*i*_ have a null probability of being on the boundary). We first extend the CNLS procedure and propose a new procedure called CMM to estimate the nonlinear mixed effect model as follows:

1. Compute *U*_α_(*x*) from Equation (3)
2. For each pollen tube *i*, estimate ***θ***_*i*_ by minimizing least squares
  $$\begin{array}{l} {\hat{\theta }}_{i}=\arg\underset{\theta_{i}}{\min}\overset{n_{i}}{\underset{j=1}{\sum }}{\left(y_{ij}-\lambda_{i}U_{\alpha }\left(\mu_{i}x_{ij}\right)\right)}^{2} \end{array}$$
  under the constraints $\Lambda^{*}\left(\theta_{i}\right)>0$ and ***θ***_*i*_ > 0
3. Estimate ***θ***_0_ by ${\hat{\theta }}_{0}=\frac{\sum_{i=1}^{m}{\hat{\theta }}_{i}}{m}$
4. Estimate σ^2^ by ${\hat{\sigma }}^{2}=\frac{\sum_{i=1}^{m}\sum_{j=1}^{n_{i}}{\left(y_{ij}-{\hat{\lambda }}_{i}U_{\alpha }\left({\hat{\mu }}_{i}x_{ij}\right)\right)}^{2}}{\sum_{i=1}^{m}\left(n_{i}-2\right)}$
5. Estimate Σ_0_ by ${\hat{\Sigma }}_{0}=\sum_{i=1}^{m}\frac{\left({\hat{\theta }}_{i}-{\hat{\theta }}_{0}\right){\left({\hat{\theta }}_{i}-{\hat{\theta }}_{0}\right)}^{T}}{m-1}-{\hat{\sigma }}^{2}\sum_{i=1}^{m}\frac{T_{i}^{-1}}{m}$, where $T_{i}={\left[\frac{\partial R_{i}}{\partial \theta_{i}^{T}}\right]}^{T}\left[\frac{\partial R_{i}}{\partial \theta_{i}^{T}}\right]{|}_{\theta_{i}={\hat{\theta }}_{i}}$ and $R_{i}={\left(R\left(x_{i1};\theta_{i}\right),R\left(x_{i2};\theta_{i}\right),\cdots \;,R\left(x_{in_{i}};\theta_{i}\right)\right)}^{T}$
6. Modify the estimator of Σ_0_ by
  $$\begin{array}{l} {\hat{\Sigma }}_{+}=\{\begin{array}{cc} {\hat{\Sigma }}_{0} & \text{if }{\hat{\Sigma }}_{0}\text{is positive definite}\\ Q{\text{Ψ}}_{+}Q^{\prime } & \text{if }{\hat{\Sigma }}_{0}\text{is not positive definite} \end{array} \end{array}$$
  where Ψ_+_ is a diagonal matrix whose diagonal elements Ψ_*ii*_ = *max*(ψ_*i*_, 0), where ψ_*i*_ is the eigenvalue of ${\hat{\Sigma }}_{0}$, and Q is a 2 × 2 matrix whose *i*^*th*^ columns is the eigenvector *q*_*i*_ associated with ψ_*i*_.
7. Convert ${\hat{\theta }}_{0}$ to ${\hat{k}}_{nf}$ and ${\hat{k}}_{pf}$.

This procedure is motivated by the MM proposed by Lu and Meeker (1993), and we extend it to the constrained case. The asymptotic normalities of the CMM estimators ${\hat{\theta }}_{0}$, ${\hat{\Sigma }}_{0}$ are proved in the Web Appendix (Proposition 6).

**Remark 4:** If *n*_*i*_ is sufficiently large, then the estimators ***θ***_*i*_ are closed enough to the true parameters so that even with a small *m*, the mean (our population estimator) will be also very close to the ***θ***_0_. In addition, because of the independence of the tubes, the quasi-normality of each estimator for big *n* implies that the population estimator will be also almost Gaussian, as a simple mean. However, if *n*_*i*_ is not large enough, then it requires sufficiently large *m* to obtain a close estimate, or (approximate) Gaussianity.

We consider now the estimator of the population parameter $\phi_{0}=\left(k_{nf}^{0},k_{pf}^{0}\right)$ defined as $k_{nf}^{0}=E_{G}\left[k_{nf}\left(\theta_{0}\right)\right]$ and $k_{pf}^{0}=E_{G}\left[k_{pf}\left(\theta_{0}\right)\right]$. Let ${\hat{\phi }}_{0}=\phi \left({\hat{\theta }}_{0}\right)$ be the estimator of ***ϕ***_0_. By the delta-method, we can prove the asymptotic normality of ${\hat{\phi }}_{0}$ (see Corollary 2 in Web Appendix).

## 3. Results

### 3.1. Simulation Studies

In this section, our simulation studies demonstrate that the proposed methods can provide robust and accurate estimates of *k*_*pf*_ and *k*_*nf*_ based on data from either single pollen tube or multiple pollen tubes, an important step toward a deeper understanding of the tip growth of normal and mutant pollen tubes. All the estimation procedures were implemented in R.

To evaluate the performance of the CNLS procedure, we simulated data using the values α = 1.2, *D*_1_ = 0.2, *R*_*tot*_ = 30, *L*_0_ = 15, *k*_*nf*_ = 0.2, and *k*_*pf*_ = 0.3. These values were obtained empirically from real wild type Arabidopisis pollen tube data, which are also similar as those in the Supplementary Table 2 in Luo et al. (2017). Accordingly, μ = 1, λ = 0.6, and $L_{0}\sqrt{k_{nf}D_{1}}$. Although our *k*_*nf*_ and *k*_*pf*_ values are different from those used in Luo et al. (2017), our ratio of 1.5 is close to their ratio of 30 * 0.028/0.5081 = 1.65. Note that our *k*_*pf*_/*k*_*nf*_ is roughly equivalent to their *k*_*pf*_/*k*_*nf*_ × *R*_*tot*_. As we mentioned earlier, it is the ratio $\frac{k_{pf}}{k_{nf}}$ that determines the height of the peak. The solution of *U*_α_ is solved by the collocation method (Bader and Ascher, 1987) in R package “bvpSolve.” Recall that *U*_α_(*x*) is a positive and even function that achieves its maximum at *x* = 0. With α = 1.2, *U*_α_(*x*) is close to $\frac{1}{2}$ at |*x*| = 5 and is close to 0 at |*x*| ≥ 15. Therefore, *R*(*x*) = λ*U*_α_(μ*x*) with μ = 1 in the simulation studies were generated from |*x*| < 15, and the range of *R*(*x*) is [0, 0.9663].

For each measurement error σ = 0.2, 0.4, we generated 10,000 data sets of size *n* = 51, 101, 301, i.e., *x* were picked along [−15, 15] with step size of 0.1, 0.3, and 0.6. CNLS based estimates of the parameters were obtained for each of the 10,000 data sets, based on which the relative bias and SD were computed as shown in Supplementary Table 1. We could see the CNLS procedure works quite well and is quite robust against noise when the size of data is fairly large. We also followed Proposition 4 to compute asymptotical variances and construct the coverage probability as shown in Supplementary Table 1. $K=E_{X}\left[\nabla_{\theta }R\left(X;\theta_{0}\right)\nabla_{\theta }R{\left(X;\theta_{0}\right)}^{T}\right]$ in Proposition 4 cannot be computed analytically. However, when *n* ≥ 300, it can be well approximated by its sample mean $\frac{1}{n}\sum_{i=1}^{n}\nabla_{\theta }R\left(x_{i};\theta_{0}\right)\nabla_{\theta }R{\left(x_{i};\theta_{0}\right)}^{T}$ according to our simulation. We could see that the asymptotical variances computed based on Proposition 4 are close to those computed based on simulation, and the observed coverage appears to be approximately equivalent to the nominal confidence level.

To evaluate the performance of the CMM procedure, we generated data for each *m* pollen tubes with associated (μ_*i*_, λ_*i*_) simulate from *MVN*((μ, λ)^*T*^, Σ). The true values of parameters used for the simulation were *k*_*nf*_ = 0.2, *k*_*pf*_ = 0.3, μ = 1, λ = 0.6, σ = 0.2, and Σ is a diagonal matrix with $\Sigma_{11}=\sigma_{\mu }^{2}=0.06^{2}$, $\Sigma_{22}=\sigma_{\lambda }^{2}=0.06^{2}$, and $\Sigma_{12}=\rho \sigma_{\lambda }\sigma_{\mu }=0.8*0.06^{2}$.

We considered three cases. In case 1, *m* = 10, *x* is uniformly sampled from –15 to 15 with step size 0.6 and *n* = 51. In case 2, *m* = 10, *x* is uniformly sampled from –15 to 15 with step size 0.3 and *n* = 101. In case 3, *m* = 50, *x* is uniformly sampled from –15 to 15 with step size 0.1 and *n* = 301. Each simulation was done 10,000 times. The relative bias, SD and coverage probability for the CMM procedure are shown in Supplementary Table 2, from which we can see that the CMM procedure performs well. In particular, the asymptotical variances computed based on Proposition 6 are close to those computed based on simulation, and the observed coverage appears to be approximately equivalent to the nominal confidence level. Similar results were also observed by Yang (2001).

### 3.2. Pollen Tube Data Analysis

#### 3.2.1. Data Preprocessing

In this section, we analyze data from real pollen tubes. We have measured the ROP1 intensities of 12 pollen tubes of Arabidopsis on a reference regular grid defined on (−10μ*m*, 10μ*m*) with a step size of 0.1205μ*m*, therefore, *m* = 12 and *n* = 173. In order to remove outliers and to reduce excessive noise while taking into account the correlations among the 12 tubes, we replace the data with their projections on the most significant axis of a Principal Component Analysis (PCA). We select 9 axes for reconstructing the data in all the tubes (instead of 12 axes for exact reconstruction), which represent 98% of the total variance, as shown in Figure 3. Nevertheless, the tubes are not observed at all the same points *x*_*j*_ of the reference grid, and the observed grid (*x*_*ij*_) may vary with tube *i*. In order to deal with the random locations (on a common grid), we consider that the tubes have missing values (around 25% of missing values for the whole dataset) and we use the R package missMDA (Josse and Husson, 2016) for computing the axis and PCA. As a by-product of the PCA, we impute also the observations at all the locations of the reference grid, for all the tubes. For simplicity, although our mixed-effect estimator is consistent for tubes with random designs, we compute the individual and population estimators with the imputed data, so that the data are defined on the same uniform grid, for all the tubes.

**Figure 6:**
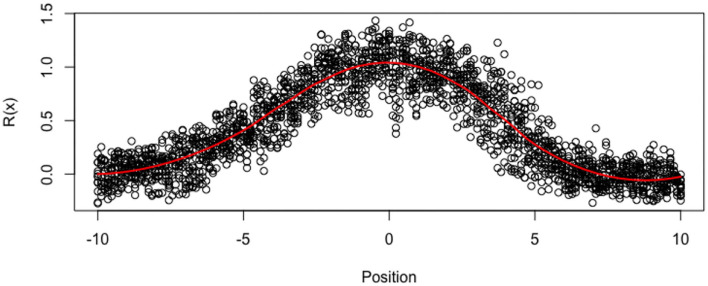
Normalized data *y*_*ij*_ for all tubes at all locations. Red line is loess smoother.

**Figure 7:**
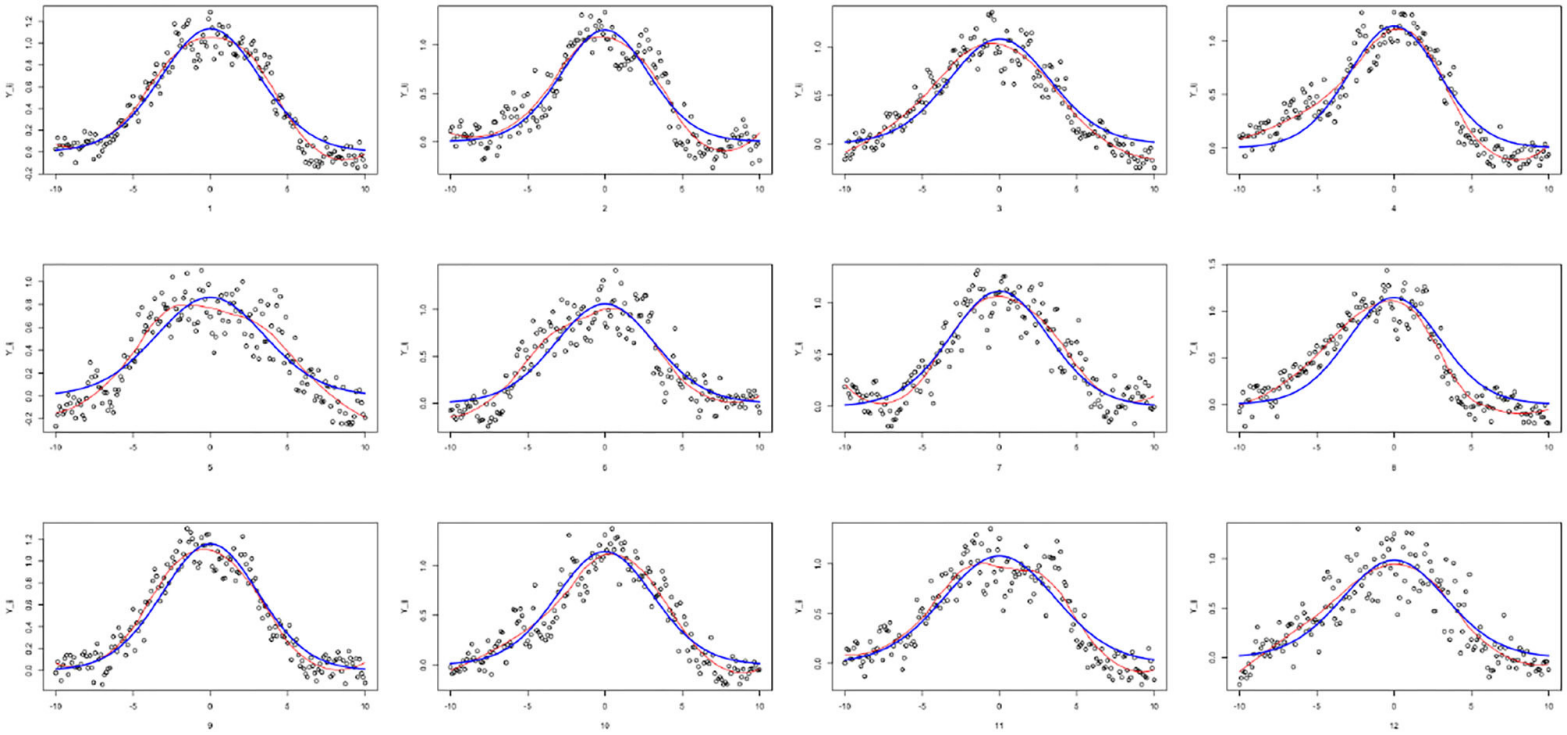
Individually fitted curves for each tube: loess-smoothed curves (in red), IDE solution *R*_λ,μ_ (in blue).

As the ROP1 intensities ỹ_*ij*_ in tubes are measured by relative intensity, it cannot represent the modeled true intensities. In particular, nonparametric smoothing estimates suggest that the functions cannot vanish at the boundaries. For this reason, we standardize the data with respect to the boundaries $y_{ij}=\frac{{ỹ}_{ij}-{\overset{-}{ỹ}}_{B}}{{\overset{-}{ỹ}}_{B}}$ (where ${\overset{-}{ỹ}}_{B}=\frac{1}{N_{B}}\sum_{i,|x_{ij}|>8}\tilde{y_{ij}}$ is the mean of the boundary values), in order to get ROP1 intensities that can vanish at the boundaries, as shown in Figure 6. The estimated SD of the noise is then 0.146. Figure 7 shows the normalized tubes and the smoothed estimate (in red) of *R*_*i*_ for *i* = 1, …, 12.

As the functions *R*_*i*_ can be written as *R*_*i*_(*x*) = λ_*i*_*U*_α_(μ_*i*_*x*), this means that any renormalization by a given constant *R*_0_ only impacts λ_*i*_. If ${\tilde{R}}_{i}=\frac{R_{i}}{R_{0}}$, then the normalized distribution has corresponding parameters $\left(\mu_{i},\frac{\lambda_{i}}{R_{0}}\right)$ and it satisfies the IDE


(16) $$\begin{array}{l} -D_{1}\partial_{x}^{2}{\tilde{R}}_{i}=-k_{nf}{\tilde{R}}_{i}+k_{pf}R_{0}^{\alpha -1}{\tilde{R}}_{i}^{\alpha }\left(1-\frac{\int {\tilde{R}}_{i}dx}{{\tilde{R}}_{total}}\right) \end{array}$$

where ${\tilde{R}}_{total}=\frac{R_{total}}{R_{0}}$, and the corresponding parameters are simply shifted in the following way $\left({\tilde{k}}_{nf},{\tilde{k}}_{pf}\right)=\left(k_{nf},k_{pf}R_{0}^{\alpha -1}\right)$, and the ratio is changed accordingly $\frac{{\tilde{k}}_{nf}}{{\tilde{k}}_{pf}}=\frac{1}{R_{0}^{\alpha -1}}\frac{k_{nf}}{k_{pf}}$. This ratio is an important quantity in the IDE as it controls the height of the peak.

Note that the Equation (2) or the normalized Equation (16) are the mathematical models we construct for the ROP1 data *y*. The difference between its solution *R*_*i*_ and *y*_*ij*_, *j* = 1, ⋯ , 173 for each *i, i* = 1, ⋯ , 12, is caused by random error as described in Equation (6) for signle pollen tube and Equation (13) for multiple pollent tube. We then apply estimation procedure detailed in Section 2.

#### 3.2.2. Estimation of Individual Parameters

Following the results of paper (Luo et al., 2017), we assume that the diffusion parameter is *D*_1_ = 0.2, the shape parameter α = 1.2, and *R*_*tot*_ = 30. Moreover, as the data does not vanish exactly at the boundaries, we select *L*_0_ = 15 which permits to deal with a function that will not be exactly 0 for *x* = ±10. For *i* = 1, …, 12, we compute a rough estimate of ${\overset{-}{R}}_{i}=\int_{L_{0}}^{L_{0}}R_{i}\left(x\right)dx$ based on the nonparametric smoother of Figure 7. We find that ${\overset{-}{R}}_{i}$ is between 7 and 9.5, i.e., the percentage of active ROP1 on the membrane is around 23% to 30% of *R*_*tot*_. For each tube, we compute the CNLS estimates of (λ_*i*_, μ_*i*_) and $k_{nf},k_{pf},\frac{k_{nf}}{k_{pf}}$, and σ (see Table 1). Table 1 suggests that although we have a non-negligible tube to tube variation, we can obtain pretty robust and consistent estimate for the ratio $\frac{k_{nf}}{k_{pf}}$ (and λ as well). This is consistent with our observation that all 12 pollen tubes have similar shapes and heights (as shown in Figure 7).

**Table 1:** The individual parameter estimates with constrained nonlinear least square (CNLS) for tubes *i* = 1, …, 12.

	**1**	**2**	**3**	**4**	**5**	**6**	**7**	**8**	**9**	**10**	**11**	**12**
μ	0.99	1.12	0.97	1.09	0.90	0.99	1.00	1.07	1.02	1.02	0.88	0.95
λ	0.70	0.72	0.67	0.71	0.54	0.66	0.69	0.71	0.72	0.71	0.67	0.61
*k*_*nf*_	0.20	0.25	0.19	0.24	0.16	0.20	0.20	0.23	0.21	0.21	0.16	0.18
*k*_*pf*_	0.31	0.37	0.29	0.35	0.25	0.30	0.31	0.34	0.32	0.32	0.25	0.28
σ	0.13	0.17	0.16	0.17	0.18	0.19	0.17	0.20	0.12	0.16	0.19	0.20
$\frac{k_{nf}}{k_{pf}}$	0.64	0.67	0.64	0.67	0.65	0.65	0.65	0.66	0.65	0.65	0.62	0.65

This suggests that estimating individual $\frac{k_{nf}}{k_{pf}}$ using each single pollen tube data can reveal whether the pollen tubes are undergoing the same mechanism of polarity formation or not and potentially discover new mutants with new mechanisms.

#### 3.2.3. Estimation of the Population Parameters

If all pollen tubes are from the same population (e.g., the 12 tubes we are analyzing in this paper), we can use the constrained mixed model's approach to obtain the population parameter estimates ${\hat{\mu }}_{0}=1.0$ (95% confidence interval is [0.96, 1.03]) and ${\hat{\lambda }}_{0}=0.625$ (95% confidence interval [0.59, 0.66]), with a SD for the noise $\hat{\sigma }=0.18$. From these estimates, we derive the population parameter estimates for ${\hat{k}}_{nf}^{0}=0.2$ (95% Confidence Interval is [0.18, 0.21]) and ${\hat{k}}_{pf}^{0}=0.3$ (95% Confidence Interval is [0.28;0.32]).

The SD in the population are ${\hat{\sigma }}_{\mu }=0.059$ and ${\hat{\sigma }}_{\lambda }=0.061$, with an estimated correlation ${\hat{\rho }}_{\mu ,\lambda }=0.81$. The estimated correlation ${\hat{\rho }}_{k_{nf},k_{pf}}=0.96$ indicates that there is a quasi-linear relationship between *k*_*nf*_, *k*_*pf*_. Above all, the mixed model permits us to estimate the correlation between the parameters and explains the strong relationship observed in Figure 5.

The multiple tube analysis and the introduction of a flexible (semi)-parametric model on the geometric properties of the densities has permitted us to infer the remarkable relationships between *k*_*nf*_ and *k*_*pf*_. We believe that this link is deeply related to the growth process of pollen tubes. In fact, in Luo et al. (2017), the mathematical model used shows that the tube geometry (tube width) is related to the values of the ratio $\frac{k_{nf}}{k_{pf}}$, and we refer to the Web Appendix of Luo et al. (2017) in which the Figure 3a relates a given ratio $\frac{k_{nf}}{k_{pf}}$ to the width of pollen tubes. Our mixed-model then gives a way to measure this link through the population covariance.

## 4. Discussion

In this paper, we propose a statistical estimation procedure for an IDE model. Such models are quite difficult to analyze and estimate in general, as the existence of a solution and the qualitative behavior can be very specific. Nevertheless, we have shown through a mathematical analysis that the space of solutions can be reparametrized in a much more efficient manner. We have derived a versatile parametrization of the solution that links the shape of the distribution *R* (described with parameters μ, λ) to the competition between positive and negative feedback loops *k*_*nf*_, *k*_*pf*_. Based on this relationship, we have derived an estimator with its complete statistical properties, by taking into account individual variability with a mixed model approach and asymptotic arguments. Thanks to the properties of CNLS, we can derive an estimator with few parametric assumptions on the distribution of the possible shapes of the densities *R*_*i*_ (we only specify the first two moments of G). Indeed, from the available data, it is arguable to assume standard parametric assumptions on G, such as a Gaussian distribution—as shown in Figure 5: in our analysis, we rely instead on the asymptotic normality of the CNLS estimators. In addition, the (degenerated) deterministic relationship between *k*_*nf*_ and *k*_*pf*_ is also very hard to introduce directly in a mixed-effects model. On the contrary, the multiple tube analysis and the introduction of a flexible (semi)-parametric model on the geometric properties of the densities has permitted to infer the remarkable relationships between *k*_*nf*_ and *k*_*pf*_. We think that this link is deeply related to the growth process of pollen tubes.

Oscillatory spatiotemporal *Ca*^2+^ signals have been observed in experiments, and it is identified as an important driving force of the polar cell growth in Arabidopsis pollen tubes (Luo et al., 2017). Tian et al. (2019) proposed a new reaction-diffusion model of ROP1 and *Ca*^2+^ interaction on the plasma membrane which incorporates *k*_*nf*_,*k*_*pf*_, *D*_1_, and *Ca*^2+^. Estimating *k*_*nf*_ and *k*_*pf*_ using ROP1 data at steady state proposed in this paper can provide reasonable initial values for *k*_*nf*_ and *k*_*pf*_ in Tian et al. (2019)'s PDE model to facilitate a more complete and realistic description of the tube geometry to have better estimates, or to introduce other characteristics of the ROP1 distribution that could be related to the trade-off between negative and positive feedbacks.

The mathematical tools (in particular the theory of semilinear diffusion equation) used for deriving the existence of a solution could provide ways for deriving a tractable parametrization of the solutions set, amenable for statistical estimation. Another challenging situation is also the presence of (existence) constraints that may be reached with real data. We give in the Web Appendix the corresponding asymptotics in that situation, but the impact of reaching such constraints during estimation may have a consequence on the validity of the model and might be used for model selection. Nevertheless, in our case, the linear constraints are far from being saturated, and we always accept the NLS estimator.

## Data Availability Statement

The data analyzed in this study is subject to the following licenses/restrictions: The dataset is available upon request. Requests to access these datasets should be directed to yang@ucr.edu.

## Author Contributions

XC and ZY initiated the project. ZX, NB, and XC developed the methods, theories, and wrote the paper. ZX and CT performed simulations. ZX and NB performed real data analysis. JG helped the real data analysis and interpretation. All authors contributed to the article and approved the submitted version.

## Funding

This work was partially supported by the United States Department of Agriculture (USDA) National Institute of Food and Agriculture (NIFA) Hatch Project AES-CE award (CA-R-STA-7132-H) (XC) and NSF DMS 185369 (XC, ZY, and JG).

## Conflict of Interest

The authors declare that the research was conducted in the absence of any commercial or financial relationships that could be construed as a potential conflict of interest.

## Publisher's Note

All claims expressed in this article are solely those of the authors and do not necessarily represent those of their affiliated organizations, or those of the publisher, the editors and the reviewers. Any product that may be evaluated in this article, or claim that may be made by its manufacturer, is not guaranteed or endorsed by the publisher.
