# Supplementary material for: Constrained Nonlinear and Mixed Effects Integral Differential Equation Models for Dynamic Cell Polarity Signaling
Source: Front Plant Sci. 2022 May 25;13:847671. doi: 10.3389/fpls.2022.847671 (PMC9175011; doi:10.3389/fpls.2022.847671)
Supplement: Supplementary file 1 [file Data_Sheet_1.PDF]

Supplementary Material: Constrained Nonlinear and Mixed Effects Differential Equation Models for Dynamic Cell Polarity Signaling

Zhen Xiao Nicolas Brunel Chenwei Tian Jingzhe Guo Zhenbiao Yang Xinping Cui

August 3, 2020

1. APPENDIX

Lemma 1. *For all $c \in [0, \infty)$, and $\alpha > 1$, there exists an unique positive solution U_α to the semilinear elliptic equation (1). Moreover, $x \mapsto U_\alpha(x)$ is positive, even and increasing at $(-c, 0]$ and decreasing at $[0, c)$. The L_1 norm $\int_{-c}^{c} U_\alpha(x)dx$ is denoted by $|U_\alpha|$.*

$$\begin{cases} -\partial_x^2 u = -u + u^\alpha, & x \in [-c, c] \\ u(-c) = u(c) = 0. \end{cases} \tag{1}$$

Proof Based on the classical theory of the differential equation, there are potentially two solutions to the semilinear elliptical equation (1) including the null solution. Therefore, to prove Lemma 1, one only needs to show that there exists a non-null solution U_α to equation (1), and $U_\alpha > 0$ on $[-c, c]$.

The existence of a positive solution to the semilinear elliptic equation $-\partial_x^2 u = f(u)$ is discussed in Lions (1982). In our case, $f(u) = u^\alpha - u$. Therefore, $f(0) = 0$, $f'(0) = -1 < 0$, and $f(u)$ is superlinear since $\frac{f(u)}{u} \to \infty$ as $u \to \infty$. By the Theorem 1.1 in Lions (1982), there exists a positive function U_α in $C^2([-c, c])$ that satisfies equation (1). Furthermore, when $c = +\infty$, the existence and uniqueness of solution to the equation (1) can also be

proved by the Theorem 1.1.3 in Cazenave and Haraux (1998). From Gidas et al. (1979), it is easy to see that U_α is a positive and even function which increases at $[-c, 0]$ and decreases at $[0, c]$.

As c tends to infinity, the solution U_α gets closer to the solution of the Dirichlet problem defined on $\mathbb{R}$, with limit conditions $u(x) \longrightarrow 0$ as $|x| \longrightarrow \infty$ (see Figure 1 in Web Appendix). In fact, when the solution is defined on $\mathbb{R}$, it has a closed-form expression $U_\alpha(x) = \left(\frac{\alpha+1}{2}\right)^{\frac{1}{\alpha-1}} \cosh\left(\frac{\alpha-1}{2}x\right)$. In addition, the influence of α is the same and monotonous, i.e., the peak is getting thinner as α increases. All these suggest a limited sensitivity of the solution U_α to α and c.

Lemma 2. *For all $\lambda' > 0$, and parameters $D_1, k_{nf}, k_{pf} > 0$, and for all $\alpha > 1$, there exists an unique positive solution $R_{\lambda'}(x)$ to the semilinear elliptic equation (2).*

$$\begin{cases} -D_1 \partial_x^2 u = -k_{nf} u + \lambda' k_{pf} u^\alpha, x \in [-L_0, L_0] \\ u(-L_0) = u(L_0) = 0 \end{cases} \tag{2}$$

Moreover, if U_α is the unique positive solution to (1) defined on $\Omega' = \left[-L_0\sqrt{\frac{k_{nf}}{D_1}}, L_0\sqrt{\frac{k_{nf}}{D_1}}\right]$, then

$$R_{\lambda'}(x) = \left(\frac{k_{pf}}{k_{nf}}\lambda'\right)^{\frac{1}{1-\alpha}} U_\alpha\left(\sqrt{\frac{k_{nf}}{D_1}}x\right) \tag{3}$$

Proof Similar as the proof of Lemma 1, one only needs to show that there exists a non-null solution $R_{\lambda,\mu}(x)$ to equation (2), and $R_{\lambda,\mu}(x) > 0$ on $[-L_0, L_0]$.

Consider a family of functions $R_{\lambda,\mu}(x) = \lambda U_\alpha(\mu x)$ where $\lambda > 0$, $\mu > 0$, and U_α is the unique positive solution to equation (1) for $c = \mu L_0$. Then,

$$\partial_x R_{\lambda,\mu} = \lambda\mu \partial_x U_\alpha(\mu x)$$
$$\partial_x^2 R_{\lambda,\mu} = \lambda\mu^2 \partial_x^2 U_\alpha(\mu x)$$

By equation (1),

$$-\partial_x^2 R_{\lambda,\mu} = \lambda^{1-\alpha}\mu^2 R_{\lambda,\mu}^\alpha - \mu^2 R_{\lambda,\mu}.$$

Therefore, $R_{\lambda,\mu}$ satisfies $-D_1\partial_x^2 R_{\lambda,\mu} = -D_1\mu^2 R_{\lambda,\mu} + D_1\lambda^{1-\alpha}\mu^2 R_{\lambda,\mu}^{\alpha}$. Since $\mu, k_{nf}, k_{pf}, D_1 > 0$, we can take $\mu = \sqrt{\frac{k_{nf}}{D_1}}$ and $\lambda = \left(\frac{k_{pf}}{k_{nf}}\lambda'\right)^{\frac{1}{1-\alpha}}$, then $-D_1\partial_x^2 R_{\lambda,\mu} = -k_{nf}R_{\lambda,\mu} + \lambda' k_{pf} R_{\lambda,\mu}^{\alpha}$. Therefore, $R_{\lambda,\mu}$ is the unique positive solution to equation (2).

Remark 1. *The parameter λ' is an instrumental parameter introduced for adding an extra degree-of-freedom in the family of solutions. Surprisingly, Lemma 2 shows that the set of solutions of equation (2) generated by the parameters $(D_1, k_{nf}, k_{pf}) \in \mathbb{R}^3$ are in fact only two-dimensional. For this reason, we introduce the 2D parametric family of functions $R_{\lambda,\mu}(x) = \lambda U_\alpha(\mu x)$, for $\lambda, \mu > 0$. The functions $R_{\lambda'}$ are a subset that corresponds to $\lambda = (\frac{k_{pf}}{k_{nf}}\lambda')^{\frac{1}{1-\alpha}}$ and $\mu = \sqrt{\frac{k_{nf}}{D_1}}$.*

Proposition 1. *Let U_α be the positive solution of (1) on $\Omega' = \left[-L_0\sqrt{\frac{k_{nf}}{D_1}}, L_0\sqrt{\frac{k_{nf}}{D_1}}\right]$. We introduce the discriminant function*

$$\Lambda(R_{tot}, D_1, \alpha, L_0, k_{nf}, k_{pf}) = \frac{k_{nf}}{k_{pf}} - \frac{1}{\alpha}\left(\frac{\alpha-1}{\alpha}\sqrt{\frac{k_{nf}}{D_1}}\frac{R_{tot}}{|U_\alpha|}\right)^{\alpha-1} \tag{4}$$

1. *If $\Lambda(R_{tot}, D_1, \alpha, L_0, k_{nf}, k_{pf}) > 0$, there is no solution to the IDE.*

2. *If $\Lambda(R_{tot}, D_1, \alpha, L_0, k_{nf}, k_{pf}) = 0$, there is a unique positive solution to the IDE. The solution can be written as $R_{\lambda,\mu}$, with $\lambda = \frac{\alpha-1}{\alpha}\frac{\mu R_{tot}}{|U_\alpha|}$ and $\mu = \sqrt{\frac{k_{nf}}{D_1}}$.*

3. *If $\Lambda(R_{tot}, D_1, \alpha, L_0, k_{nf}, k_{pf}) < 0$, there are two positive solutions to the IDE. The two solutions can be written as $R_{\lambda,\mu}$, with $\mu = \sqrt{\frac{k_{nf}}{D_1}}$ and λ being the root of the following equation*

$$\frac{k_{nf}}{k_{pf}} - \lambda^{\alpha-1} + \lambda^{\alpha}\sqrt{\frac{D_1}{k_{nf}}}\frac{|U_\alpha|}{R_{tot}} = 0. \tag{5}$$

Proof of Sufficient Condition Since $R_{\lambda'}(x) = R_{\lambda,\mu}(x)$ is a solution to (2), $R_{\lambda'}(x)$ is also a solution to IDE if $\lambda' = \frac{k_{nf}}{k_{pf}}\lambda^{1-\alpha} = \left(1 - \frac{1}{R_{tot}}|R_{\lambda'}|\right)$, where $|R_{\lambda'}| = \int_{-L_0}^{L_0} R_{\lambda,\mu}(x)dx = \lambda\sqrt{\frac{D_1}{k_{nf}}}|U_\alpha|$. Denote $g(\lambda) \doteq \frac{k_{nf}}{k_{pf}} - \lambda^{\alpha-1} + \frac{1}{R_{tot}}\lambda^{\alpha}\sqrt{\frac{D_1}{k_{nf}}}|U_\alpha|$, then $g'(\lambda) = \lambda^{\alpha-2}\left(-(\alpha-1) + \frac{\alpha}{R_{tot}}\sqrt{\frac{D_1}{k_{nf}}}|U_\alpha|\lambda\right)$. The root λ_c of $g'(\lambda)$ is $\lambda_c = \frac{\alpha-1}{\alpha}\sqrt{\frac{k_{nf}}{D_1}}\frac{R_{tot}}{|U_\alpha|}$. $g(\lambda)$ is decreasing in $[0, \lambda_c]$ and increasing in

$[\lambda_c, +\infty]$ since $g'(\lambda) = \lambda^{\alpha-2}(\alpha-1)(\frac{\lambda}{\lambda_c} - 1)$. Notice that $g(0) = \frac{k_{nf}}{k_{pf}}$, $\lim_{+\infty} g = +\infty$, and

$$g(\lambda_c) = \frac{k_{nf}}{k_{pf}} - \frac{1}{\alpha}\left(\frac{\alpha-1}{\alpha}\sqrt{\frac{k_{nf}}{D_1}}\frac{R_{tot}}{|U_\alpha|}\right)^{\alpha-1}$$

1. When $g(\lambda_c) > 0$, $g(\lambda) > 0$, no positive solution to IDE can be found from the family of solutions to (2).

2. When $g(\lambda_c) = 0$, $g(\lambda) > 0$ for $\lambda \neq \lambda_c$, therefore one positive solution $R_{\lambda_c,\mu}(x)$ to IDE can be found from the family of solutions to (2).

3. When $g(\lambda_c) < 0$, there exist $\lambda_1 \in [0, \lambda_c]$ and $\lambda_2 \in [\lambda_c, \infty]$ such that $g(\lambda_1) = 0$ and $g(\lambda_2) = 0$, therefore two positive solutions $R_{\lambda_1,\mu}(x)$ and $R_{\lambda_2,\mu}(x)$ to IDE can be found from the family of solutions to (2).

Proof of Necessary Condition It is only necessary to show that for any positive solution R of IDE on $[-L_0, L_0]$, there exist $\lambda, \mu > 0$ such that $U_\alpha(x) = \frac{1}{\lambda}R(\frac{x}{\mu})$ is a solution to (1) on $\left[-\frac{L_0}{\mu}, \frac{L_0}{\mu}\right]$. Denote $\bar{\lambda} = \frac{1}{\lambda}$, $\bar{\mu} = \frac{1}{\mu}$, then $\frac{\partial U_\alpha(x)}{\partial x} = \bar{\lambda}\bar{\mu}\frac{\partial R(\bar{\mu}x)}{\partial(\bar{\mu}x)}$ and $\frac{\partial^2 U_\alpha(x)}{\partial x^2} = \bar{\lambda}\bar{\mu}^2\frac{\partial^2 R(\bar{\mu}x)}{\partial(\bar{\mu}x)^2}$. $U_\alpha(x)$ is a solution to (1) on $[-L_0\bar{\mu}, L_0\bar{\mu}]$ if and only if

$$\begin{aligned}
&-\frac{\partial^2 U_\alpha(x)}{\partial x^2} = -U_\alpha(x) + U_\alpha^\alpha(x)\\
&-\bar{\lambda}\bar{\mu}^2 R''(\bar{\mu}x) = -\bar{\lambda}R(\bar{\mu}x) + \bar{\lambda}^\alpha R^\alpha(\bar{\mu}x)\\
&-\bar{\mu}^2\frac{k_{nf}R(\bar{\mu}x)}{D_1} + \bar{\mu}^2\frac{k_{pf}R^\alpha(\bar{\mu}x)}{D_1}\left(1 - \frac{\int_{\frac{L_0}{\bar{\mu}}}^{\frac{L_0}{\bar{\mu}}} R(\bar{\mu}x)d\bar{\mu}x}{R_{tot}}\right) = -R(\bar{\mu}x) + \bar{\lambda}^{\alpha-1}R^\alpha(\bar{\mu}x)
\end{aligned}$$

when $\bar{\mu} = \sqrt{\frac{D_1}{k_{nf}}}$, $\bar{\lambda}$ can be obtained by solving the equality

$$\frac{k_{pf}}{k_{nf}}R^\alpha(\bar{\mu}x)\left(1 - \frac{\int_{\frac{L_0}{\bar{\mu}}}^{\frac{L_0}{\bar{\mu}}} R(\bar{\mu}x)d\bar{\mu}x}{R_{tot}}\right) = \bar{\lambda}^{\alpha-1}R^\alpha(\bar{\mu}x)$$

for which $\bar{\lambda} = \left[\frac{k_{pf}}{k_{nf}}\left(1 - \frac{\int_{-L_0}^{L_0} R(x)dx}{R_{tot}}\right)\right]^{\frac{1}{\alpha-1}}$. Hence, $\bar{\lambda}$ exists if and only if $\frac{\int_{-L_0}^{L_0} R(x)dx}{R_{tot}} < 1$. Suppose $\frac{\int_{-L_0}^{L_0} R(x)dx}{R_{tot}} \geq 1$, then the right hand side of IDE is nonpositive and therefore the left

hand side of IDE must be nonpositive. That is, $R''(x) > 0$. Therefore, $R(x)$ must be a convex function. This is impossible because $R(x)$ is a positive function with $R(-L_0) = R(L_0) = 0$. Therefore, $\frac{\int_{-L_0}^{L_0} R(x)dx}{R_{tot}} < 1$ always holds for $R(x) > 0$ and $\bar{\lambda}$ exists, which completes the proof.

Remark 2. *For $\lambda > 0$, the solution $R_{\lambda,\mu}(x)$ to IDE is a positive and even function. Moreover, it increases on $[-L_0, 0]$ and decreases on $[0, L_0]$. Consequently the maximum is located at $x = 0$, and $\max_{x\in\Omega} R_{\lambda,\mu}(x) = R_{\lambda,\mu}(0) = \lambda U_\alpha(0) > \lambda$. The proof is provided below. It will be shown later in section 6 that the ROP1 data in real-world experiments reflects these qualitative properties.*

Proof By Lemma 1, $U_\alpha > 0$ and even which increases at $[-c, 0]$ and decreases at $[0, c]$. Therefore, the maximum of U_α should be located at $x = 0$. Moreover, in the proof of Lemma 1, $-\partial_x^2 U_\alpha|_{x=0} = U_\alpha^\alpha(0) - U_\alpha(0) > 0$. Note that the function $f(x) = x^\alpha - x$ is such that $f(1) = 0$, and $f(x) > 0$ for $x > 1$. Therefore, $U_\alpha(0) > 1$. As a result, $\max_x R_{\lambda,\mu}(x) = \lambda U_\alpha(0) > \lambda$.

Remark 3. *From the sufficient condition, we see that the solution to IDE is determined by two parameters, μ and λ, which can be viewed as a reparametrization of k_{nf} and k_{pf}. In other words, IDE is not over parametrized by k_{nf} and k_{pf} given fixed values of D_1,R_{tot} and L_0.*

Proposition 2. *Let $R(x; k_{nf}, k_{pf})$ denote the solution to IDE. $R(x; .)$ is a positive and non-constant function on interval $[-L_0, L_0]$. $R(x; k_{nf}^0, k_{pf}^0) = R(x; k_{nf}^1, k_{pf}^1)$ on $[-L_0, L_0]$ if and only if $k_{pf}^0 = k_{pf}^1$ and $k_{nf}^0 = k_{nf}^1$.*

Proof Suppose for parameters (k_{nf}^0, k_{pf}^0) and (k_{nf}^1, k_{pf}^1), $R(x; k_{nf}^0, k_{pf}^0) = R(x; k_{nf}^1, k_{pf}^1)$ on $[-L_0, L_0]$, then we have

$$k_{nf}^0 R(x) - k_{pf}^0 R^\alpha(x) \left(1 - \frac{\int_{-L_0}^{L_0} R(x)dx}{R_{tot}}\right) = k_{nf}^1 R(x) - k_{pf}^1 R^\alpha(x) \left(1 - \frac{\int_{-L_0}^{L_0} R(x)dx}{R_{tot}}\right)$$

For $R(x) > 0$ on $[-L_0, L_0]$

$$k_{nf}^0 - k_{nf}^1 = (k_{pf}^0 - k_{pf}^1) R^{\alpha-1}(x) \left(1 - \frac{\int_{-L_0}^{L_0} R(x)dx}{R_{tot}}\right)$$

If $k_{pf}^0 - k_{pf}^1 \neq 0$, then

$$R^{\alpha-1}(x) = (k_{nf}^0 - k_{nf}^1) / \left((k_{pf}^0 - k_{pf}^1) \left(1 - \frac{\int_{-L_0}^{L_0} R(x)dx}{R_{tot}} \right) \right),$$

suggesting that $R(x)$ has to be constant on $[-L_0, L_0]$ since $1 - \frac{\int_{-L_0}^{L_0} R(x)dx}{R_{tot}} > 0$. By contradiction, we can show that $k_{pf}^0 - k_{pf}^1 = 0$ and $k_{nf}^0 - k_{nf}^1 = 0$, i.e., $R(x; k_{nf}^0, k_{pf}^0) = R(x; k_{nf}^1, k_{pf}^1)$ on $[-L_0, L_0]$ if and only if $k_{pf}^0 = k_{pf}^1$ and $k_{nf}^0 = k_{nf}^1$.

Proposition 3. *The constrained nonlinear model*

$$Y_j = R(X_j; k_{nf}, k_{pf}) + \epsilon_j \qquad j = 1, 2, \cdots, n. \tag{6}$$

can be reparametrized into the following model with μ and λ subject to the linear constraints

$$Y_j = \lambda U_\alpha(\mu X_j) + \epsilon_j, \qquad j = 1, 2, \cdots, n, \tag{7}$$

$$\begin{cases} \Lambda^*(\mu, \lambda) = \mu R_{tot} - \lambda |U_\alpha| > 0 \\ \lambda > 0 \\ \mu > 0 \end{cases} \tag{8}$$

where $\mu = \sqrt{\frac{k_{nf}}{D_1}}$ and λ is the root of $g(\lambda)$.

Proof We just need to check that the discriminant Λ is negative. In order to do this, we need to show the following function $h(\mu, \lambda)$ is non-positive.

Lemma: For any $\mu > 0$ and $\lambda > 0$, the function $h(\mu, \lambda) = \lambda^{\alpha-1} - \lambda^\alpha \frac{|U_\alpha|}{\mu R_{tot}} - \frac{1}{\alpha} \left(\frac{\alpha-1}{\alpha} \frac{\mu R_{tot}}{|U_\alpha|} \right)^{\alpha-1}$ is always non-positive.

Proof. For any fixed $\mu > 0$, h is a function of λ whose first-order derivative is 0 if and only if $\lambda \doteq \lambda_c = \frac{\alpha-1}{\alpha} \frac{\mu R_{tot}}{|U_\alpha|}$. Then, we have

$$h(\mu, \lambda_c) = \lambda_c^{\alpha-1} - \lambda_c^\alpha \frac{|U_\alpha|}{\mu R_{tot}} - \frac{1}{\alpha} \left(\frac{\alpha-1}{\alpha} \frac{\mu R_{tot}}{|U_\alpha|} \right)^{\alpha-1} = 0$$

$$h(\mu, 0) = -\frac{1}{\alpha} \left(\frac{\alpha-1}{\alpha} \frac{\mu R_{tot}}{|U_\alpha|} \right)^{\alpha-1} < 0$$

$$h(\mu, +\infty) = -\infty < 0$$

Notice that $h(\mu, \lambda)$ is a continuous function of λ, we can conclude that $h(\mu, \lambda) \leq 0$ based on the above three equations. Therefore, the Lemma holds.

When the constraints in model (6) are satisfied, from Proposition 1, $\frac{k_{nf}}{k_{pf}} = \lambda^{\alpha-1} - \frac{1}{R_{tot}}\lambda^{\alpha}\frac{1}{\mu}|U_\alpha| > 0$ and $\mu > 0$ and $\lambda > 0$. Therefore, the constraints in model (7) hold. When the constraints in model (7) are satisfied, we can convert μ and λ to k_{nf} and k_{pf} by solving $k_{nf} = D_1\mu^2$ and $\frac{k_{nf}}{k_{pf}} = \lambda^{\alpha-1} - \frac{1}{R_{tot}}\lambda^{\alpha}\frac{1}{\mu}|U_\alpha|$. The solution of k_{nf} and k_{pf} is such that $k_{nf} > 0$, $k_{pf} > 0$ and by Lemma 1, $\Lambda(k_{nf}, k_{pf}) = \frac{k_{nf}}{k_{pf}} - \frac{1}{\alpha}(\frac{\alpha-1}{\alpha}\sqrt{\frac{k_{nf}}{D_1}}\frac{R_{tot}}{|U_\alpha|})^{\alpha-1} = \lambda^{\alpha-1} - \lambda^{\alpha}\frac{|U_\alpha|}{R_{tot}\mu} - \frac{1}{\alpha}\left(\frac{\alpha-1}{\alpha}\frac{R_{tot}\mu}{|U_\alpha|}\right)^{\alpha-1} \leq 0$. Therefore, the constraints in model (6) hold.

Proposition 4. *Let $R(X;\boldsymbol{\theta}) = \lambda U_\alpha(\mu X)$, if $\boldsymbol{\theta}_0$ is not on the boundary then*

$$\sqrt{n}(\hat{\boldsymbol{\theta}}_n - \boldsymbol{\theta}_0) \xrightarrow{d} \mathcal{N}(0, \sigma^2 K^{-1})$$

where $K = E_X[\nabla_{\boldsymbol{\theta}} R(X;\boldsymbol{\theta}_0)\nabla_{\boldsymbol{\theta}} R(X;\boldsymbol{\theta}_0)^T]$, and $\nabla_{\boldsymbol{\theta}} R(X;\boldsymbol{\theta}_0)$ is the gradient vector of $R(X;\boldsymbol{\theta})$ with respect to $\boldsymbol{\theta}$ at $\boldsymbol{\theta} = \boldsymbol{\theta}_0$. If $\boldsymbol{\theta}_0$ is on the boundary,

$$\sqrt{n}(\hat{\boldsymbol{\theta}}_n - \boldsymbol{\theta}_0) \xrightarrow{d} \mathcal{CN}(\sigma^2 K^{-1})$$

Proof: Let $A = (a_{ij})_{2\times 2}$ denote a symmetric two by two matrix. Suppose all four entries of A are bounded in $[-b, b]$ for some $b > 0$. For any vector $\boldsymbol{x} = (x_1, x_2)^T$, $\boldsymbol{x}^T A\boldsymbol{x} = a_{11}x_1^2 + 2a_{12}x_1x_2 + a_{22}x_2^2 \leq 2bx_1^2 + 2bx_2^2 = 2b\boldsymbol{x}^T\boldsymbol{x}$. Therefore, $A \leq 2bI_2$.

Denote $z = (z_\mu, z_\lambda)^T = n^{1/2}(\boldsymbol{\theta} - \boldsymbol{\theta_0})$. It can be easily seen that minimizing (9) under the constraints (10)

$$(\hat{\lambda}, \hat{\mu}) = \arg\min_{\lambda,\mu}\sum_{j=1}^{n}(y_j - \lambda U_\alpha(\mu x_j))^2 \tag{9}$$

$$\begin{cases} \Lambda^*(\mu,\lambda) = \mu R_{tot} - \lambda\,|U_\alpha| > 0 \\ \lambda > 0 \\ \mu > 0 \end{cases} \tag{10}$$

is equivalent to

$$\begin{aligned} &\min_z \sum_{j=1}^n \{[\epsilon_j + R(x_j, \boldsymbol{\theta_0}) - R(x_j, \boldsymbol{\theta_0} + n^{-1/2}z)]^2 - \epsilon_j^2\} \\ \texttt{s.t.}\, &g_1(\boldsymbol{\theta_0} + n^{-1/2}z) = -(\mu_0 + n^{-1/2}z_\mu)R_{tot} + (\lambda_0 + n^{-1/2}z_\lambda)|U_\alpha| < 0 \\ &g_2(\boldsymbol{\theta_0} + n^{-1/2}z) = -(\mu_0 + n^{-1/2}z_\mu) < 0 \\ &g_3(\boldsymbol{\theta_0} + n^{-1/2}z) = -(\lambda_0 + n^{-1/2}z_\lambda) < 0 \end{aligned} \tag{11}$$

where ϵ_j are i.i.d with $N(0, \sigma^2)$.

Assume the optimal solution to (11) exists and denote it by $\hat{z}_n$. Then $\hat{z}_n = n^{1/2}(\hat{\boldsymbol{\theta}}_n - \boldsymbol{\theta}_0)$. To prove proposition 4, we need to discuss if the true parameter is on the boundary or not. The proof is essentially based on two steps (and is essentially the same in both cases). We focus on the case where θ_0 is not on the boundary. First, we prove that the limit of (11) is

$$\texttt{min}\ z'Kz - 2z'\xi \tag{12}$$

when $n \to \infty$, where $\xi \sim N(0, \sigma^2 K)$. Then, we prove the solution to (11) converges in distribution to the solution to (12).

Step 1: Limit problem of (11)

Denote the objective function $F_n(\epsilon, z) = \sum_{j=1}^n \{[\epsilon_j + R(x_j, \boldsymbol{\theta_0}) - R(x_j, \boldsymbol{\theta_0} + n^{-1/2}z)]^2 - \epsilon_j^2\}$ and parameter space $S_n = \{z : g_1(\boldsymbol{\theta_0} + n^{-1/2}z) < 0, g_2(\boldsymbol{\theta_0} + n^{-1/2}z) < 0, g_3(\boldsymbol{\theta_0} + n^{-1/2}z) < 0\}$. To formulate the limit problem of (11), we have the following results.

Result 1: When $\sigma^2 = 1$, for each fixed $z \in \mathbb{R}^2$, $F_n(\epsilon, z)$ converges in distribution to $F(\xi, z) = z'Kz - 2z'\xi$, where $\xi \sim N(0, K)$.

(i) As specified above, $\epsilon_1, \epsilon_2, \ldots, \epsilon_n$ are *i.i.d.* with $E(\epsilon_i) = 0$ and $Var(\epsilon_i) = \sigma^2 = 1$.

(ii) $R(x_j; \boldsymbol{\theta}) = \lambda U_\alpha(\mu x_j)$, $j = 1, \ldots, n$, are differentiable in $\boldsymbol{\theta}$ since $U_\alpha(\mu x_j)$ is differentiable in μ. By Taylor expansion,

$$R(x_j; \boldsymbol{\theta}) = R(x_j; \boldsymbol{\theta}_0) + (\nabla_{\boldsymbol{\theta}} R(x_j; \boldsymbol{\theta}_0))^T(\boldsymbol{\theta} - \boldsymbol{\theta}_0) + \frac{1}{2}(\boldsymbol{\theta} - \boldsymbol{\theta}_0)^T \Delta_{\boldsymbol{\theta}} R(x_j; \boldsymbol{\theta}_0)(\boldsymbol{\theta} - \boldsymbol{\theta}_0) + o(\|\boldsymbol{\theta} - \boldsymbol{\theta}_0\|^2)$$

where $\nabla_{\boldsymbol{\theta}} R(x_j; \boldsymbol{\theta_0}) = \begin{pmatrix} \lambda_0 x_j U'_\alpha(\mu_0 x_j) \\ U_\alpha(\mu_0 x_j) \end{pmatrix}$ and $\triangle_{\boldsymbol{\theta}} R(x_j; \boldsymbol{\theta_0}) = \begin{pmatrix} \lambda_0 x_j^2 U''_\alpha(\mu_0 x_j) & x_j U'_\alpha(\mu_0 x_j) \\ x_j U'_\alpha(\mu_0 x_j) & 0 \end{pmatrix}$.

Let $r_j(\boldsymbol{\theta}) = \{R(x_j;\boldsymbol{\theta}) - R(x_j;\boldsymbol{\theta_0}) - \bigtriangledown_{\boldsymbol{\theta}} R(x_j;\boldsymbol{\theta_0})^T(\boldsymbol{\theta}-\boldsymbol{\theta_0})\}/\parallel \boldsymbol{\theta}-\boldsymbol{\theta_0} \parallel^2$. Since $R(x_j;\boldsymbol{\theta})$, $\bigtriangledown_{\boldsymbol{\theta}} R(x_j;\boldsymbol{\theta_0})^T(\boldsymbol{\theta}-\boldsymbol{\theta_0})$,$\parallel \boldsymbol{\theta}-\boldsymbol{\theta_0} \parallel^2$ are continuous on $\boldsymbol{\theta}$, $r_j(\boldsymbol{\theta})$ is a continuous function on $\boldsymbol{\theta}$.

It's obvious that there exists $b > 0$ such that all elements in $\triangle_{\boldsymbol{\theta}} R(x_j, \boldsymbol{\theta_0})$ are bounded by $[-b, b]$. Therefore, we have

$$\begin{aligned} |r_j(\boldsymbol{\theta})| \parallel \boldsymbol{\theta}-\boldsymbol{\theta}_0 \parallel^2 &= |\frac{1}{2}(\boldsymbol{\theta}-\boldsymbol{\theta}_0)^T \Delta_{\boldsymbol{\theta}} R(x_j;\boldsymbol{\theta}_0)(\boldsymbol{\theta}-\boldsymbol{\theta}_0) + o(\|\boldsymbol{\theta}-\boldsymbol{\theta}_0\|^2)| \\ &\leq \frac{1}{2}(\boldsymbol{\theta}-\boldsymbol{\theta}_0)^T 2bI_2(\boldsymbol{\theta}-\boldsymbol{\theta}_0) \\ &= b\|\boldsymbol{\theta}-\boldsymbol{\theta}_0\|^2 \end{aligned}$$

Therefore, $|r_j(\boldsymbol{\theta})| \leq b$ and $\lim_{n\to\infty} \frac{1}{n}\sum_{j=1}^n r_j^2(\boldsymbol{\theta}) \leq b^2 < \infty$ holds in the whole parameter space.

(iii) Since $\bigtriangledown_{\boldsymbol{\theta}} R(x_j, \boldsymbol{\theta}_0)\bigtriangledown_{\boldsymbol{\theta}} R(x_j, \boldsymbol{\theta}_0)' = \begin{pmatrix} \lambda_0^2 x_j^2 U_\alpha'(\mu_0 x_j)^2 & \lambda_0 x_j U_\alpha(\mu_0 x_j) U_\alpha'(\mu_0 x_j) \\ \lambda_0 x_j U_\alpha(\mu_0 x_j) U_\alpha'(\mu_0 x_j) & U_\alpha(\mu_0 x_j)^2 \end{pmatrix}$, all the elements in $\bigtriangledown_{\boldsymbol{\theta}} R(x_j, \boldsymbol{\theta}_0) \bigtriangledown_{\boldsymbol{\theta}} R(x_j, \boldsymbol{\theta}_0)'$ are bounded. By Kolmogorov's Strong Law of Large Numbers (SLLN), we have

$$\frac{1}{n}\sum_{j=1}^{n}[\bigtriangledown_{\boldsymbol{\theta}} R(x_j, \boldsymbol{\theta}_0)(\bigtriangledown_{\boldsymbol{\theta}} R(x_j, \boldsymbol{\theta}_0))'] \xrightarrow{a.s.} K$$

where

$$K = \begin{pmatrix} E_X[\lambda_0^2 X^2 U_\alpha'(\mu_0 X)^2] & E_X[\lambda_0 X U_\alpha(\mu_0 X) U_\alpha'(\mu_0 X)] \\ E_X[\lambda_0 X U_\alpha(\mu_0 X) U_\alpha'(\mu_0 X)] & E_X[U_\alpha(\mu_0 X)^2] \end{pmatrix}$$

By Cauchy-Schwarz inequality we have

$$det(K) = E_X[\lambda_0^2 X^2 U_\alpha'(\mu_0 X)^2] E_X[U_\alpha(\mu_0 X)^2] - (E_X[\lambda_0 X U_\alpha(\mu_0 X) U_\alpha'(\mu_0 X)])^2 \geq 0$$

However, if equality holds, it implies that $\lambda_0 X U_\alpha'(\mu_0 X)$ and $U_\alpha(\mu_0 X)$ are linearly dependent, i.e., there exists a non-zero scalar $a \in R$ such that $\lambda_0 X U_\alpha'(\mu_0 X) = aU_\alpha(\mu_0 X)$ holds for any value of X since $U_\alpha(\mu_0 X)$ and $U_\alpha'(\mu_0 X)$ are both continuously differentiable with respect to X. As a result, $U_\alpha(\mu_0 X)$ is a solution to the linear ODE $\lambda_0 X u'(X) - au(X) = 0$. This would imply that U_α is proportional to some power function $x \mapsto x^{a'}$, which contradicts its own characteristics as shown in Lemma 1. Therefore $det(K) > 0$. Since $trace(K) > 0$, both eigenvalues of K must be positive. Therefore K is positive definite.

Therefore, $\lim_{n\to\infty} \frac{1}{n} \sum_{j=1}^{n} [\bigtriangledown_{\boldsymbol{\theta}} R(x_j, \boldsymbol{\theta}_0)(\bigtriangledown_{\boldsymbol{\theta}} R(x_j, \boldsymbol{\theta}_0))'] = K$ exists and is positive definite. And by Theorem 1 of Wang (1996), $F_n(\epsilon, z)$ converges in distribution to $F(\xi, z) = z'Kz - 2z'\xi$.

Result 2: It is obvious that $g_i(\boldsymbol{\theta}), i = 1, 2, 3$, are continuously differentiable and there exists no equality constraints. Also because

$$\begin{cases} g_1(\boldsymbol{\theta}_0) = \mu_0 R_{tot} - \lambda_0 |U_\alpha| \neq 0 \\ g_2(\boldsymbol{\theta}_0) = \mu_0 \neq 0 \\ g_3(\boldsymbol{\theta}_0) = \lambda_0 \neq 0 \end{cases} .$$

I is an empty set. Therefore, by theorem 2 of Wang (1996), we have parameter space S_n converges in Kuratowski's sense to S, the parameter space of (12). Combining **Result 1** and **Result 2**, the limit problem of (11) is minimizing $z'Kz - 2z'\xi$ without constraint.

Step 2 Convergence property of solution to (11)

According to Theorems 3-6 of Wang (1996), the solution to the limit problem (12) should be unique at $B(M) = z :\| z \| < M$ for any large M, so that the solution to (11) converges in distribution to the solution to (12).

Since the limit problem (12) is minimizing $z'Kz - 2z'\xi$ without constraint, there is a unique solution $z = K^{-1}\xi$ at $B(M) = z :\| z \| < M$ for any $M >\| K^{-1}\xi \|$. Therefore, by Theorems 3-6 of Wang (1996), $\hat{z}_n$ of the limit problem (11) converges in distribution to $z = K^{-1}\xi \sim N(0, \sigma^2 K^{-1})$, i.e. $\hat{z}_n = \sqrt{n}(\hat{\theta}_n - \theta_0) \xrightarrow{d} N(0, \sigma^2 K^{-1})$. This completes the proof.

For any $\sigma^2 > 0$, based on Theorems 4 and 5 of Jennrich (1969),

$$n^{-\frac{1}{2}} \sum_{i=1}^{n} \nabla_{\boldsymbol{\theta}} R(X_i; \boldsymbol{\theta}_0) \boldsymbol{\epsilon}_i \xrightarrow{d} N(0, \sigma^2 K)$$

As a result, $F_n(\boldsymbol{\epsilon}_i, \boldsymbol{z})$ will converge in distribution to $\boldsymbol{z}'K\boldsymbol{z} - 2\boldsymbol{z}'\boldsymbol{\xi}$ where $\boldsymbol{\xi}$ is a random vector following $N(0, \sigma^2 K)$. In fact, based on Theorems 1-5 of Jennrich (1969), Theorems 1-6 of Wang (1996) still hold even if σ^2 is unknown.

Finally, we consider the situation when $\boldsymbol{\theta}_0$ is on the boundary, meaning that $\mu R_{tot} - \lambda|U_\alpha| = 0$. The big difference is that the constraint is asymptotically active. With exactly the same argument as before, we can apply Theorem 6 of Wang (1996), and the limit problem is

$$\begin{cases} \min_z z'Kz - 2z'\xi \\ z'a \leq 0 \end{cases} \tag{13}$$

The solution to (13) is solved with Lagrangian relaxation, in the context of minimizing a convex (positive quadratic form) function under a linear constraint. Consequently, the Karush-Kuhn-Tucker (KTT) conditions give exactly the solution, with which we can derive the law below.

Per KKT conditions, there exists $z^*, \mu^* \geq 0$ such that

$$\begin{cases} \nabla_z Q(z^*) + \mu^* a = 0 \\ \mu^* a' z^* = 0 \end{cases}$$

This implies two cases:

- if the constraint is not active, i.e $a'z^* < 0$, then $\mu^* = 0$ and the condition is

$$\nabla_z Q(z) = 0$$

$$2Kz - 2\xi = 0$$

  The matrix K is positive definite, and we have $z^* = K^{-1}\xi$.

- If the constraint is active, we have $a'z^* = 0$ and $\mu^* > 0$, and we need to solve the equation

$$2Kz^* - 2\xi + \mu^* a = 0$$

  This means that we have $z^* = K^{-1}\left(\xi - \frac{\mu^*}{2}a\right)$ and we must satisfy the orthogonality constraint $a'z^* = 0$ which is equivalent to

$$a'K^{-1}\left(\xi - \frac{\mu^*}{2}a\right) = 0$$

$$a'K^{-1}\xi = \frac{\mu^*}{2}a'K^{-1}a$$

This implies that the Lagrangian multiplier is

$$\mu^* = 2\frac{a'K^{-1}\xi}{a'K^{-1}a}$$

and

$$\begin{aligned} z^* &= K^{-1}\left(\xi - \frac{a'K^{-1}\xi}{a'K^{-1}a}a\right) \\ &= K^{-1}\left(I_m - \frac{aa'}{a'K^{-1}a}K^{-1}\right)\xi \end{aligned}$$

If we denote $Q_a = \left(I_m - \frac{aa'}{a'K^{-1}a}K^{-1}\right)$ (orthogonal projection on the complementary of a with respect to the inner product K^{-1}), then z^* inherits a Gaussian distribution from ξ, and $z^* \sim \mathcal{N}\left(0, \sigma_\epsilon^2 K^{-1} Q_a K Q_a K^{-1}\right)$.

The solution in the constrained case is

$$\begin{cases} a'K^{-1}\xi < 0, & z^* = K^{-1}\xi \\ a'K^{-1}\xi \geq 0, & z^* = K^{-1}Q_a\xi \end{cases}$$

Consequently, the distribution of the solution z^* is a kind of mixture of two Gaussians $\mathcal{N}\left(0, \sigma_\epsilon^2 K^{-1}\right)$ (if no constraints) and $\mathcal{N}\left(0, \sigma_\epsilon^2 K^{-1} Q_a K Q_a K^{-1}\right)$ if there is a constraint, which behaves as the Gaussian distribution $N(0, \sigma^2 K^{-1})$ on the half plan $a'K^{-1}z < 0$, and behaves as the degenerate Gaussian distribution $N\left(0, \sigma_\epsilon^2 K^{-1} Q_a K Q_a K^{-1}\right)$ on the half-plane $a'K^{-1}z \geq 0$. The corresponding distribution is denoted $\mathcal{CN}(\sigma^2 K^{-1})$ in the paper.

The estimation of the standard deviation is straightforward, as shown in the next proposition.

Proposition 5. *If $\boldsymbol{\theta}_0$ is not on the boundary, define the estimator of σ^2 as $\hat{\sigma}_n^2 = \frac{1}{n-2}\sum_{j=1}^n \left(y_j - \hat{\lambda}U_\alpha(\hat{\mu}x_j)\right)^2$, then*

$$\hat{\sigma}_n^2 \xrightarrow{p} \sigma^2$$

By Slutsky's theorem

$$\frac{\sqrt{n}(\hat{\boldsymbol{\theta}}_n - \boldsymbol{\theta}_0)}{\hat{\sigma}_n} \xrightarrow{d} \mathcal{N}(0, K^{-1})$$

Proof

$$\hat{\sigma}_n^2 = \frac{1}{n-2}\sum_{j=1}^{n}(y_j - R(x_j,\hat{\boldsymbol{\theta}}_n))^2 = \frac{1}{n-2}\sum_{j=1}^{n}(R(x_j,\boldsymbol{\theta}_0)+\epsilon_j - R(x_j,\hat{\boldsymbol{\theta}}_n))^2$$
$$= \frac{1}{n-2}\sum_{j=1}^{n}(R(x_j,\boldsymbol{\theta}_0) - R(x_j,\hat{\boldsymbol{\theta}}_n))^2 + \frac{2}{n-2}\sum_{j=1}^{n}(R(x_j,\boldsymbol{\theta}_0) - R(x_j,\hat{\boldsymbol{\theta}}_n))\epsilon_j + \frac{1}{n-2}\sum_{j=1}^{n}\epsilon_j^2 \tag{14}$$

First, we prove that $\frac{1}{n-2}\sum_{j=1}^{n}(R(x_j,\boldsymbol{\theta}_0) - R(x_j,\hat{\boldsymbol{\theta}}_n))^2 \xrightarrow{p} 0$. From proof of Proposition 4, we have $\frac{1}{n-2}\sum_{j=1}^{n}\bigtriangledown_{\boldsymbol{\theta}}R(x_j,\boldsymbol{\theta}_0)\bigtriangledown_{\boldsymbol{\theta}}R(x_j,\boldsymbol{\theta}_0)^T \xrightarrow{a.s.} K$ and $K \leq 2bI$. Since $\sqrt{n}(\hat{\boldsymbol{\theta}}_n-\boldsymbol{\theta}_0) \xrightarrow{d} N(0,\sigma^2K^{-1})$, we have $\hat{\boldsymbol{\theta}}_n \xrightarrow{L_2} \boldsymbol{\theta}_0$. Therefore, we have

$$\begin{aligned}&\frac{1}{n-2}\sum_{j=1}^{n}(R(x_j,\boldsymbol{\theta}_0) - R(x_j,\hat{\boldsymbol{\theta}}_n))^2\\ &= (\hat{\boldsymbol{\theta}}_n-\boldsymbol{\theta}_0)^T[\frac{1}{n-2}\sum_{j=1}^{n}\bigtriangledown_{\boldsymbol{\theta}}R(x_j,\boldsymbol{\theta}_0)\bigtriangledown_{\boldsymbol{\theta}}R(x_j,\boldsymbol{\theta}_0)^T](\hat{\boldsymbol{\theta}}_n-\boldsymbol{\theta}_0) + o(\parallel \hat{\boldsymbol{\theta}}_n-\boldsymbol{\theta}_0 \parallel^2)\\ &\xrightarrow{p} 0\end{aligned} \tag{15}$$

Since $\bigtriangledown_{\boldsymbol{\theta}}R(x_j,\boldsymbol{\theta}_0) = \begin{pmatrix}\lambda_0 x_j U'_\alpha(\mu_0 x_j)\\ U_\alpha(\mu_0 x_j)\end{pmatrix}$ which is continuous and bounded, by theorem 4 of Jennrich (1969), we have $\frac{1}{n-2}\sum_{j=1}^{n}\bigtriangledown_{\boldsymbol{\theta}}R(x_j,\boldsymbol{\theta}_0)\epsilon_j \xrightarrow{p} 0$. Therefore, we have

$$\frac{1}{n-2}\sum_{j=1}^{n}(R(x_j,\boldsymbol{\theta}_0)-R(x_j,\hat{\boldsymbol{\theta}}_n))\epsilon_j = (\hat{\boldsymbol{\theta}}_n-\boldsymbol{\theta}_0)^T\{\frac{1}{n-2}\sum_{j=1}^{n}\bigtriangledown_{\boldsymbol{\theta}}R(x_j,\boldsymbol{\theta}_0)\epsilon_j\}+o(\parallel \hat{\boldsymbol{\theta}}_n-\boldsymbol{\theta}_0 \parallel) \xrightarrow{p} 0 \tag{16}$$

By SLLN, $\frac{1}{n}\sum_{j=1}^{n}\epsilon_j^2 \xrightarrow{a.s.} \sigma^2$. From equation (14), (15) and (16), we have $\hat{\sigma}_n^2 \xrightarrow{p} \sigma^2$.

Denote $\boldsymbol{\phi} = (k_{nf}, k_{pf})^\top$, $\boldsymbol{\phi}_0 = (k_{nf}(\boldsymbol{\theta}_0), k_{pf}(\boldsymbol{\theta}_0))^\top$ to be the true value of $\boldsymbol{\phi}$, and $\hat{\boldsymbol{\phi}}_n$ to be the estimator of $\boldsymbol{\phi}$ based on

$$\begin{cases}\hat{k}_{nf} = D_1\hat{\mu}^2\\ \hat{k}_{pf} = \frac{D_1\hat{\mu}^2}{\hat{\lambda}^{\alpha-1}-\frac{\hat{\lambda}^{\alpha}|U_\alpha|}{\hat{\mu}R_{tot}}}\end{cases} \tag{17}$$

By using the delta method, the asymptotic distribution of $\hat{\boldsymbol{\phi}}_n$ is derived as shown in Corollary 1.

Corollary 1. *If $\boldsymbol{\theta}_0$ is not on the boundary, then $\hat{\boldsymbol{\phi}}_n$ is asymptotically normal, as below:*

$$\frac{\sqrt{n}(\hat{\boldsymbol{\phi}}_n - \boldsymbol{\phi}_0)}{\hat{\sigma_n}} \xrightarrow{d} N(0, A^T K^{-1} A)$$

where

$$A = \begin{pmatrix} \frac{\partial k_{nf}}{\partial \mu} & \frac{\partial k_{pf}}{\partial \mu} \\ \frac{\partial k_{nf}}{\partial \lambda} & \frac{\partial k_{pf}}{\partial \lambda} \end{pmatrix} = \begin{pmatrix} 2D_1\mu & \frac{2D_1\mu^3 - 3D_1\mu^2\lambda\frac{|U_\alpha|}{R_{tot}}}{\lambda^{\alpha-1}(\mu-\lambda\frac{|U_\alpha|}{R_{tot}})^2} \\ 0 & \frac{-D_1\mu^4(\alpha-1)+D_1\mu^3\alpha\lambda\frac{|U_\alpha|}{R_{tot}}}{\lambda^{\alpha}(\mu-\lambda\frac{|U_\alpha|}{R_{tot}})^2} \end{pmatrix}$$

Proposition 6. *Assume that*

1. *the parameter $\boldsymbol{\theta}_0$ is not on the boundary,*

2. *the sample size from each pollen tubes are equal, i.e., $n_1 = n_2 = \cdots = n_m = n$,*

3. *both n and m tend to $+\infty$.*

Then, we have the following large sample properties for $\hat{\boldsymbol{\theta}}_0$

1. $\hat{\boldsymbol{\theta}}_0 \xrightarrow{p} \boldsymbol{\theta}_0$

2. $\sqrt{m}\tilde{\Sigma}^{-\frac{1}{2}}(\hat{\boldsymbol{\theta}}_0 - \boldsymbol{\theta}_0) \xrightarrow{d} \boldsymbol{Z}$, *where* $\boldsymbol{Z} \sim N(0, I_2)$, *and* $\tilde{\Sigma} = \Sigma_0 + \sigma^2 E_{\boldsymbol{\mathcal{G}}}[(nK_i)^{-1}]$ *with* $K_i = E_X[\nabla_{\boldsymbol{\mathcal{G}}} R(X; \boldsymbol{\theta}_i) \nabla_{\boldsymbol{\mathcal{G}}} R(X; \boldsymbol{\theta}_i)^T]$.

Moreover, if $\hat{\sigma}^2$ is a consistent estimator of σ^2, then

1. $\hat{\Sigma}_0 \xrightarrow{p} \Sigma_0$

2. $\sqrt{m}\hat{\tilde{\Sigma}}^{-\frac{1}{2}}(\hat{\boldsymbol{\theta}}_0 - \boldsymbol{\theta}_0) \xrightarrow{d} \boldsymbol{Z}$, *where* $\hat{\tilde{\Sigma}} = \hat{\Sigma}_0 + \hat{\sigma}^2 E_{\boldsymbol{\mathcal{G}}}[(nK_i)^{-1}]$.

The covariance matrix defined as $\tilde{\Sigma} = \Sigma_0 + \sigma^2 E_{\boldsymbol{\mathcal{G}}}[(nK)^{-1}]$ corresponds to the total variance of the estimator of $\hat{\boldsymbol{\theta}}_i$ taking into account both the within-subject and between-subject variability.

Proof Since $\hat{\boldsymbol{\theta}}_i = (\hat{\mu}_i, \hat{\lambda}_i)^T$ is obtained by CNLS for each single pollen tube, from Proposition 4 we have,

$$\sqrt{n}(\hat{\boldsymbol{\theta}}_i - \boldsymbol{\theta}_i) \xrightarrow{d} MVN\left(\mathbf{0}, \sigma^2 (K_i)^{-1}\right)$$

for each given $\boldsymbol{\theta}_i$, where $K_i = E_X[\nabla_{\boldsymbol{\theta}} R(X; \boldsymbol{\theta}_i) \nabla_{\boldsymbol{\theta}} R(X; \boldsymbol{\theta}_i)^T]$. Since $V(\boldsymbol{\theta}_i) = \Sigma_0$, the unconditional asymptotic mean and variance of $\hat{\boldsymbol{\theta}}_i$ are

$$\begin{aligned} E(\hat{\boldsymbol{\theta}}_i) &= E_{\mathcal{G}}[E_\epsilon(\hat{\boldsymbol{\theta}}_i | \boldsymbol{\theta}_i)] \to E_{\mathcal{G}}(\boldsymbol{\theta}_i) = \boldsymbol{\theta}_0 \\ Var(\hat{\boldsymbol{\theta}}_i) &= Var_{\mathcal{G}}[E_\epsilon(\hat{\boldsymbol{\theta}}_i | \boldsymbol{\theta}_i)] + E_{\mathcal{G}}[Var_\epsilon(\hat{\boldsymbol{\theta}}_i | \boldsymbol{\theta}_i)] \to Var_{\mathcal{G}}[\boldsymbol{\theta}_i] + E_{\mathcal{G}}[(nK_i)^{-1}\sigma^2] \\ &= \Sigma_0 + \sigma^2 E_{\mathcal{G}}[(nK_i)^{-1}] \doteq \tilde{\Sigma} \end{aligned}$$

Therefore, $\{\hat{\boldsymbol{\theta}}_i : i = 1, \cdots, m\}$ are *i.i.d.* with common asymptotic mean and variance. Since $\hat{\boldsymbol{\theta}}_0 = m^{-1} \sum_{i=1}^m \hat{\boldsymbol{\theta}}_i$, from SLLN and CLT, we have

$$\begin{aligned} \frac{1}{m-1} \sum_{i=1}^m (\hat{\boldsymbol{\theta}}_i - \hat{\boldsymbol{\theta}}_0)(\hat{\boldsymbol{\theta}}_i - \hat{\boldsymbol{\theta}}_0)^T &\xrightarrow{p} \tilde{\Sigma} \\ \hat{\boldsymbol{\theta}}_0 &\xrightarrow{p} \boldsymbol{\theta}_{\mathbf{0}} \\ \sqrt{m}\tilde{\Sigma}^{-\frac{1}{2}}(\hat{\boldsymbol{\theta}}_0 - \boldsymbol{\theta}_{\mathbf{0}}) &\xrightarrow{d} \boldsymbol{Z} \end{aligned}$$

with $\boldsymbol{Z} \sim N(0, I_2)$.

Furthermore, we have

$$\begin{aligned} E(T_i^{-1}) &= E_{\mathcal{G}}[E_\epsilon(T_i^{-1} | \boldsymbol{\theta}_i)] = E_{\mathcal{G}}[E_\epsilon(\left(\left[\frac{\partial \boldsymbol{R}_i}{\partial \boldsymbol{\theta}_i^T}\right]^T \left[\frac{\partial \boldsymbol{R}_i}{\partial \boldsymbol{\theta}_i^T}\right] |_{\boldsymbol{\theta}_i = \hat{\boldsymbol{\theta}}_i} \right)^{-1} | \boldsymbol{\theta}_i)] \\ &\xrightarrow{p} E_{\mathcal{G}}\left[\left(\left[\frac{\partial \boldsymbol{R}_i}{\partial \boldsymbol{\theta}_i^T}\right]^T \left[\frac{\partial \boldsymbol{R}_i}{\partial \boldsymbol{\theta}_i^T}\right] \right)^{-1} \right] = E_{\mathcal{G}}\left[\left(\sum_{j=1}^n \left[\frac{\partial R(X_{ij}; \boldsymbol{\theta}_i)}{\partial \boldsymbol{\theta}_i^T}\right] \left[\frac{\partial R(X_{ij}; \boldsymbol{\theta}_i)}{\partial \boldsymbol{\theta}_i^T}\right]^T \right)^{-1} \right] \\ &\xrightarrow{p} E_{\mathcal{G}}[\left(nE_X[\nabla_{\boldsymbol{\theta}} R(X; \boldsymbol{\theta}_i) \nabla_{\boldsymbol{\theta}} R(X; \boldsymbol{\theta}_i)^T]\right)^{-1}] = E_{\mathcal{G}}[(nK_i)^{-1}] \end{aligned}$$

The first "$\xrightarrow{p}$" in the above equation holds since $\hat{\boldsymbol{\theta}}_i \xrightarrow{p} \boldsymbol{\theta}_i$. The second "$\xrightarrow{p}$" holds by SLLN of X. Therefore, $\{T_i^{-1} : i = 1, \cdots, m\}$ are *i.i.d.* (same distribution, and locations x_{ij} are independent) with the same asymptotic mean, and so by SLLN we have that $\frac{1}{m} \sum_{i=1}^m T_i^{-1} \xrightarrow{p} E_{\mathcal{G}}[(nK_i)]$. In addition, it's assumed that $\hat{\sigma}^2 \xrightarrow{p} \sigma^2$. Therefore, by *Slutsky's* Theorem, $\hat{\Sigma}_0 \xrightarrow{p} \tilde{\Sigma} - \sigma^2 E_{\boldsymbol{\mathcal{G}}}[(nK)^{-1}] = \Sigma_0$.

Based on the asymptotical result of $\hat{\Sigma}_0$, we know that $\hat{\tilde{\Sigma}} = \hat{\Sigma}_0 + \hat{\sigma}^2 E_{\boldsymbol{\mathcal{G}}}[(nK_i)^{-1}] \xrightarrow{p} \tilde{\Sigma}$. We also have proved that $\hat{\boldsymbol{\theta}}_0$, $\sqrt{m}(\hat{\boldsymbol{\theta}}_0 - \boldsymbol{\theta}_0) \xrightarrow{d} \tilde{\Sigma}^{\frac{1}{2}}\boldsymbol{Z}$. Therefore, by *Slutsky's* Theorem we have $\sqrt{m}\hat{\tilde{\Sigma}}^{-\frac{1}{2}}(\hat{\boldsymbol{\theta}}_0 - \boldsymbol{\theta}) \xrightarrow{d} \boldsymbol{Z}$. This completes the proof of Proposition 6.

Corollary 2. *Let $\boldsymbol{\phi}_0$ be the population mean parameter, and $\hat{\boldsymbol{\phi}}_0 = \boldsymbol{\phi}(\hat{\boldsymbol{\theta}}_0)$ be the estimator . By the delta-method,*

$$\sqrt{m}(A^T\hat{\tilde{\Sigma}}A)^{-\frac{1}{2}}(\hat{\boldsymbol{\phi}}_0 - \boldsymbol{\phi}_0) \xrightarrow{d} \boldsymbol{Z}$$

where A is given in Corollary 1.

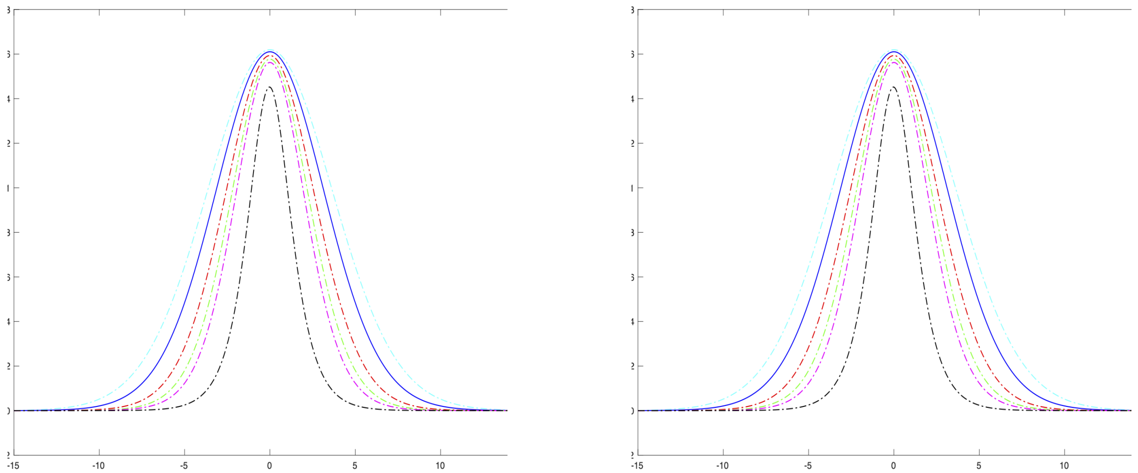

Figure 1: The solutions U_α on the whole line $\mathbb{R}$ (left panel) and on the interval $[-15, 15]$ (right panel), for $\alpha = 1.1, 1.2, 1.3, 1.4, 1.5, 2.5$. An increasing α gives a thinner peak. The solutions on $\mathbb{R}$ scarcely differ from the solutions obtained on the large interval $[-15, 15]$. The reference value in the paper is $\alpha = 1.2$ (plain blue line).

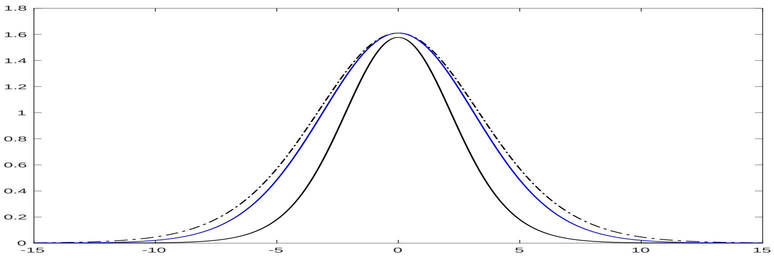

Figure 2: The solutions U_{α_1} and U_{α_2} for $\alpha_1 = 1.4$ (black, solid line) and $\alpha_2 = 1.2$ (blue, solid line) can be made similar to each other by selecting an appropriate μ in $x \mapsto U_{\alpha_1}(\mu x)$. Here, the function $x \mapsto U_{\alpha_1}(2x/3)$ (black, dashed line) is very similar to $x \mapsto U_{\alpha_2}(x)$.

REFERENCES

Cazenave, T. and Haraux, A. (1998). *An introduction to semilinear evolution equations*, volume 13 of *Oxford Lecture Series in Mathematics and its Applications*. The Clarendon Press Oxford University Press, New York.

Gidas, B., Ni, W., and Nirenberg, L. (1979). Symmetry and related properties via the maximum principle. *Communications in Mathematical Physics* **68,** 209–243.

Jennrich, R. I. (1969). Asymptotic Properties of Non-Linear Least Squares Estimators. *The Annals of Mathematical Statistics* **40,** 633–643.

Lions, P. L. (1982). On the existence of positive solutions of semilinear elliptic equations. *SIAM Review* **24,** 441–467.

Wang, J. (1996). Asymptotics of Least-Squares Estimators for Constrained Nonlinear Regression. *The Annals of Statistics* **24,** 1316–1326.

Table 1: CNLS estimators. sd: estimated standard deviation; sd^*: theoretical standard deviation based on proposition 3.

<table>
<tr><th></th><th></th><th></th><th>$\hat{k}_{nf}$</th><th>$\hat{k}_{pf}$</th><th>$\hat{\mu}$</th><th>$\hat{\lambda}$</th><th>$\hat{\sigma}_\epsilon$</th></tr>
<tr><td rowspan="12">$\sigma = 0.2$</td><td rowspan="4">n=51</td><td>bias(10^{-3})</td><td>3.75</td><td>4.65</td><td>4.33</td><td>2.03</td><td>-1.00</td></tr>
<tr><td>sd</td><td>0.0411</td><td>0.0529</td><td>0.1004</td><td>0.0497</td><td>0.0202</td></tr>
<tr><td>sd^*</td><td>0.0396</td><td>0.0512</td><td>0.0977</td><td>0.0496</td><td>0.0202</td></tr>
<tr><td>cp</td><td>0.933</td><td>0.934</td><td>0.935</td><td>0.943</td><td></td></tr>
<tr><td rowspan="4">n=101</td><td>bias(10^{-3})</td><td>1.75</td><td>2.17</td><td>1.94</td><td>0.95</td><td>-0.47</td></tr>
<tr><td>sd</td><td>0.0282</td><td>0.0363</td><td>0.0697</td><td>0.0349</td><td>0.0143</td></tr>
<tr><td>sd^*</td><td>0.0278</td><td>0.0359</td><td>0.0690</td><td>0.0352</td><td>0.0143</td></tr>
<tr><td>cp</td><td>0.941</td><td>0.942</td><td>0.942</td><td>0.952</td><td></td></tr>
<tr><td rowspan="4">n=301</td><td>bias(10^{-3})</td><td>0.87</td><td>1.11</td><td>1.41</td><td>0.60</td><td>-0.065</td></tr>
<tr><td>sd</td><td>0.0158</td><td>0.0204</td><td>0.0394</td><td>0.0201</td><td>0.0082</td></tr>
<tr><td>sd^*</td><td>0.0160</td><td>0.0207</td><td>0.0399</td><td>0.0204</td><td>0.0082</td></tr>
<tr><td>cp</td><td>0.951</td><td>0.951</td><td>0.952</td><td>0.954</td><td></td></tr>
<tr><td rowspan="12">$\sigma = 0.4$</td><td rowspan="4">n=51</td><td>bias(10^{-3})</td><td>$1.85 * 10^{-2}$</td><td>$2.25 * 10^{-2}$</td><td>$2.13 * 10^{-2}$</td><td>8.76</td><td>-2.09</td></tr>
<tr><td>sd</td><td>0.1056</td><td>0.1309</td><td>0.2222</td><td>0.1017</td><td>0.0403</td></tr>
<tr><td>sd^*</td><td>0.0857</td><td>0.1093</td><td>0.2000</td><td>0.0997</td><td>0.0404</td></tr>
<tr><td>cp</td><td>0.910</td><td>0.916</td><td>0.919</td><td>0.938</td><td></td></tr>
<tr><td rowspan="4">n=101</td><td>bias(10^{-3})</td><td>7.95</td><td>9.83</td><td>9.39</td><td>4.10</td><td>-0.96</td></tr>
<tr><td>sd</td><td>0.0608</td><td>0.0776</td><td>0.1445</td><td>0.0705</td><td>0.0285</td></tr>
<tr><td>sd^*</td><td>0.0575</td><td>0.0740</td><td>0.1396</td><td>0.0705</td><td>0.0285</td></tr>
<tr><td>cp</td><td>0.926</td><td>0.930</td><td>0.934</td><td>0.948</td><td></td></tr>
<tr><td rowspan="4">n=301</td><td>bias(10^{-3})</td><td>3.04</td><td>3.82</td><td>4.41</td><td>-1.85</td><td>-0.13</td></tr>
<tr><td>sd</td><td>0.0324</td><td>0.0418</td><td>0.0797</td><td>0.0403</td><td>0.0165</td></tr>
<tr><td>sd^*</td><td>0.0324</td><td>0.0419</td><td>0.0801</td><td>0.0408</td><td>0.0165</td></tr>
<tr><td>cp</td><td>0.948</td><td>0.949</td><td>0.949</td><td>0.953</td><td></td></tr>
</table>

Table 2: CMM estimators. *sd*: estimated standard deviation; sd^*: theoretical standard deviation; *cp*: coverage probability.

	Case	$\hat{k}_{nf}$	$\hat{k}_{pf}$	$\hat{\mu}$	$\hat{\lambda}$	$\hat{\sigma}_\epsilon$	$\hat{\sigma}_\mu$	$\hat{\sigma}_\lambda$
1	bias(10^{-3})	2.30	2.99	5.05	1.93	−0.14	−3.56	−3.28
	sd	0.0150	0.0197	0.0372	0.0246	0.0064	0.0404	0.0240
	sd^*	0.0152	0.0199	0.0378	0.0245	0.0064	0.0406	0.0242
	cp	0.936	0.928	0.941	0.930			
2	bias(10^{-3})	1.15	1.51	2.45	1.04	−0.027	−5.18	−2.36
	sd	0.0115	0.0154	0.0287	0.0217	0.0045	0.0306	0.0192
	sd^*	0.0117	0.0158	0.0292	0.0217	0.0045	0.0310	0.0194
	cp	0.938	0.938	0.940	0.929			
3	bias(10^{-3})	0.30	0.40	0.7.0	0.31	−0.002	−0.72	−0.36
	sd	0.0041	0.0055	0.0101	0.0090	0.0012	0.0088	0.0067
	sd^*	0.0041	0.0059	0.0102	0.0089	0.0012	0.0088	0.0067
	cp	0.944	0.958	0.945	0.944			
